# Non-invasive stimulation of the human striatum disrupts reinforcement learning of motor skills

**DOI:** 10.1038/s41562-024-01901-z

**Published:** 2024-05-29

**Authors:** Pierre Vassiliadis, Elena Beanato, Traian Popa, Fabienne Windel, Takuya Morishita, Esra Neufeld, Julie Duque, Gerard Derosiere, Maximilian J. Wessel, Friedhelm C. Hummel

**Affiliations:** 1 Defitech Chair of Clinical Neuroengineering, Neuro-X Institute, École Polytechnique Fédérale de Lausanne (EPFL), Geneva, Switzerland; 2 Defitech Chair of Clinical Neuroengineering, Neuro-X Institute, EPFL Valais, Clinique Romande de Réadaptation, Sion, Switzerland; 3 Institute of Neuroscience, Université Catholique de Louvain, Brussels, Belgium; 4 Foundation for Research on Information Technologies in Society, Zurich, Switzerland; 5 Lyon Neuroscience Research Center, Impact Team, Inserm U1028, CNRS UMR5292, Lyon 1 University, Bron, France; 6 Department of Neurology, University Hospital Würzburg, Würzburg, Germany; 7 Clinical Neuroscience, University of Geneva Medical School, Geneva, Switzerland

**Keywords:** Reward, Motor control

## Abstract

Reinforcement feedback can improve motor learning, but the underlying brain mechanisms remain underexplored. In particular, the causal contribution of specific patterns of oscillatory activity within the human striatum is unknown. To address this question, we exploited a recently developed non-invasive deep brain stimulation technique called transcranial temporal interference stimulation (tTIS) during reinforcement motor learning with concurrent neuroimaging, in a randomized, sham-controlled, double-blind study. Striatal tTIS applied at 80 Hz, but not at 20 Hz, abolished the benefits of reinforcement on motor learning. This effect was related to a selective modulation of neural activity within the striatum. Moreover, 80 Hz, but not 20 Hz, tTIS increased the neuromodulatory influence of the striatum on frontal areas involved in reinforcement motor learning. These results show that tTIS can non-invasively and selectively modulate a striatal mechanism involved in reinforcement learning, expanding our tools for the study of causal relationships between deep brain structures and human behaviour.

## Abstract (web summary)

Vassiliadis et al. use transcranial temporal interference stimulation—a non-invasive deep brain stimulation technique—to show that stimulation of the striatum applied at 80 Hz disrupts the ability to learn from reinforcement feedback.

## Main

The ability to learn from past outcomes, often referred to as reinforcement learning, is fundamental for complex biological systems^1^. Reinforcement learning has been classically studied in the context of decision-making, when agents have to decide among a discrete number of potential options^2^. There is increasing recognition that reinforcement learning processes are also at play in other contexts, including during practice of a new motor skill^3–5^. For instance, the addition of reinforcement feedback during motor training can improve motor learning, presumably by boosting the retention of newly acquired motor memories^6,7^. Interestingly, reinforcement feedback also appears to be relevant for the rehabilitation of patients suffering from motor impairments^8–10^. Yet, despite these promising results, there is currently a limited understanding of the brain mechanisms that are critical to implement this behaviour.

A prominent hypothesis in the field is that the striatum, a structure that is particularly active during both reinforcement^11^ and motor learning^12^, may be causally involved in the beneficial effects of reinforcement on motor learning. The striatum shares dense connections with dopaminergic structures of the midbrain as well as with prefrontal and motor cortical regions^13^ and is therefore well positioned to mediate reinforcement motor learning^14–16^. This idea is supported by neuroimaging studies showing reward-related activation of the striatum during motor learning^17,18^. More specifically, within the striatum, oscillatory activity in specific frequency bands is suggested to be involved in aspects of reinforcement processing. Previous rodent studies have shown that striatal high gamma oscillations (~80 Hz) transiently increase following reward delivery^19–23^, but not when reward is withheld^19^. Hence, dynamic changes of high gamma activity in the striatum^19,24,25^ and in other parts of the basal ganglia^26,27^ may encode the outcome of previous movements (that is, success or failure) and support learning. Consistent with a role of such oscillatory activity in reinforcement learning, high gamma activity in the striatum shows coherence with frontal cortex oscillations and is upregulated by dopaminergic agonists^19^. This body of work thus suggests that reinforcement-related modulation of striatal oscillatory activity, especially in the gamma range, may be crucial for reinforcement learning of motor skills. Conversely, striatal beta oscillations (~20 Hz) have been largely associated with sensorimotor functions^28^. For instance, beta oscillations in the striatum are exacerbated in Parkinson’s disease and associated with the severity of motor symptoms^29–31^. Consistently, excessive beta connectivity is reduced by anti-Parkinsonian treatment in proportion to the related motor improvement^32^. Taken together, these elements suggest that striatal high gamma and beta activity may have different functional roles preferentially associated with reinforcement and sensorimotor functions, respectively.

The studies mentioned above provide associative evidence linking the presence of reinforcement with changes of neural activity in the striatum determined through neuroimaging^17,18^, but they do not allow us to draw conclusions regarding its causal role in reinforcement motor learning in humans. The only causal evidence available to date comes from animal work showing modulation of reinforcement-based decision-making with striatal stimulation^33,34^. A reason for the current absence of investigations of the causal role of the striatum in human behaviour is related to its deep localization in the brain. Current non-invasive brain stimulation techniques, such as transcranial magnetic stimulation and classical transcranial electric stimulation, do not allow the selective targeting of deep brain regions, because these techniques exhibit a steep depth–focality trade-off^35,36^. Studies of patients with striatal lesions^37,38^ or invasive deep brain stimulation of connected nuclei^39,40^ have provided insights into the role of the basal ganglia in reinforcement learning. However, their conclusions are partially limited by the fact that the studied patients also exhibit altered network properties resulting from the underlying pathology (for example, neurodegeneration or lesions) or from the respective compensatory mechanisms. Here we address these challenges by exploiting transcranial temporal interference stimulation (tTIS), a recently introduced non-invasive electric brain stimulation approach allowing us to target deep brain regions in a frequency-specific and focal manner in the physiological state^41,42^.

The concept of tTIS was initially proposed and validated on the hippocampus of rodents^41^ and was then further tested through computational modelling^43–47^ and in first applications on cortical areas in humans^48,49^. tTIS requires two pairs of electrodes to be placed on the head, each pair delivering a high-frequency alternating current. One key element is that this frequency has to be high enough (that is, in the kHz range) to avoid direct neuronal entrainment, owing to the low-pass-filtering properties of neuronal membranes^50^. The second key element is the application of a small difference of frequency between the two alternating currents. The superposition of the electric fields creates an envelope oscillating at this low-frequency difference, which can be steered towards individual deep brain structures (for example, by optimizing electrodes’ placement) and is in a range able to influence neuronal activity^41,51–53^. An interesting feature of tTIS is the ability to stimulate at a particular frequency of interest to preferentially interact with specific neuronal processes^41,42^. Despite these exciting opportunities, current evidence for tTIS-related neuromodulation of deep brain structures, such as the striatum, remains sparse in humans^52,53^.

Here we combine tTIS with electric field modelling for target localization, behavioural data and functional magnetic resonance imaging (fMRI) to evaluate the causal role of specific patterns of striatal activity in reinforcement learning of motor skills. On the basis of the studies mentioned above, we hypothesized that striatal tTIS at high gamma frequency (tTIS_80Hz_) would disturb the fine-tuning of high gamma oscillatory activity in the striatum and thereby would perturb reinforcement motor learning, in contrast to beta (tTIS_20Hz_) or sham (tTIS_Sham_) stimulation. More specifically, we reasoned that applying a constant high gamma rhythm in the striatum would disturb the temporally precise and reinforcement-specific modulation of high gamma activity. Moreover, given that the stimulation protocol was not individualized to endogenous high gamma activity and not synchronized to ongoing activity in other hubs of the reinforcement learning network (for example, the frontal cortex), we anticipated disruptive rather than beneficial effects of tTIS_80Hz_.

In line with our prediction, we report that tTIS_80Hz_ disrupted motor learning compared with the controls, but only in the presence of reinforcement. To evaluate the potential neural correlates of these behavioural effects, we measured blood-oxygen-level-dependent (BOLD) activity in the striatum and effective connectivity between the striatum and frontal cortical areas involved in reinforcement motor learning. We found that the disruptive effect of tTIS_80Hz_ on reinforcement learning was associated with a specific modulation of BOLD activity in the putamen and caudate, but not in the cortex, supporting the ability of tTIS to selectively modulate striatal activity without affecting overlying cortical areas. Moreover, tTIS_80Hz_ also increased the neuromodulatory influence of the striatum on frontal cortical areas involved in reinforcement motor learning. Overall, the present study shows that tTIS can non-invasively and selectively modulate a striatal mechanism involved in reinforcement learning.

## Results

**Fig. 1:**
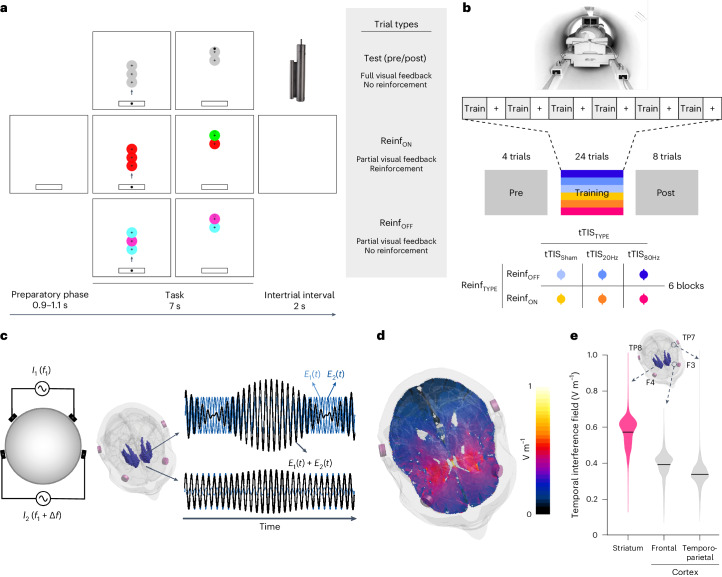
Striatal tTIS during reinforcement learning of motor skills in the MRI machine. **a**, Motor learning task. The participants were required to squeeze a hand-grip force sensor (depicted in the upper right corner of the figure) to track a moving target (the larger circle with a cross in the centre) with a cursor (the smaller black circle)^54,55^. Pre- and post-training assessments were performed with full visual feedback of the cursor and no reinforcement. In Reinf_ON_ and Reinf_OFF_ trials, the participants practised the task with or without reinforcement feedback, respectively. In Reinf_ON_ trials, the colour of the target varied in real time as a function of the participants’ tracking performance. **b**, Experimental procedure. The participants performed the task in the MRI machine with concomitant tTIS. Blocks of training were composed of 36 trials (4 pre-training, 24 training and 8 post-training trials) interspersed with short resting periods (represented as plus signs in the figure). The six training types resulted from the combination of three tTIS_TYPES_ and two Reinf_TYPES_. **c**, Concept of tTIS. On the left, two pairs of electrodes are shown on a head model, and currents *I*_1_ and *I*_2_ are applied with frequencies *f*_1_ and *f*_1_ + Δ*f*. On the right, the interference of the two electric fields within the brain is represented for two different locations with high and low envelope modulation. *E*_1_(*t*) and *E*_2_(*t*) represent the modulation of the fields’ magnitude over time. tTIS was delivered with a Δ*f* of 20 or 80 Hz or as a sham (a ramp-up and immediate ramp-down of high-frequency currents with a flat envelope). **d**, Electric field modelling with the striatal montage. The colours show the temporal interference exposure (electric field modulation magnitude). **e**, Temporal interference exposure in the striatum and in the overlying cortex. The violin plots show the tTIS exposure distribution over the voxels in the striatum and cortex underneath the stimulation electrodes. The magnitude of the field in the cortex was extracted from the BNA^64^ regions underneath the stimulation electrodes (F3–F4 and TP7–TP8). The black bar represents the mean. Voxels with outlying tTIS exposure (±5 s.d. around the mean) were removed from the plot (21 values from a total of 46,479 considered voxels).

A total of 24 healthy participants (15 women, 25.3 ± 0.1 years old (mean ± s.e.)) performed a force-tracking task in the MRI scanner with concurrent tTIS of the striatum. The task required the participants to modulate the force applied on a hand-grip force sensor to track a moving target with a cursor with the right, dominant hand^54,55^ (Fig. 1a). In each block, the participants had to learn a new pattern of motion of the target (Supplementary Fig. 1a and Methods). In Reinf_ON_ blocks, the participants were provided with online reinforcement feedback during training, giving them real-time information about success or failure throughout the trial, indicated as a green or red target, respectively (please see Supplementary Video 1 for the task). The reinforcement feedback was delivered according to a closed-loop schedule^8^, in which the success criterion to consider a force sample as successful was updated on the basis of the median performance over the four previous trials (see Methods for more details). In Reinf_OFF_ blocks, the participants practised with visually matched random feedback (cyan/magenta). Importantly, in both types of blocks, training was performed with partial visual feedback of the cursor, a condition that has been shown to maximize reinforcement effects in various motor learning paradigms^5,56–58^ and that yielded significant effects of reinforcement on motor learning, as also demonstrated in an additional behavioural study testing another group of healthy participants on the same task (*n* = 24; Supplementary Fig. 1b–e). Before and after training, the participants performed pre- and post-training assessments with full visual feedback, no reinforcement and no tTIS, allowing us to evaluate motor learning. To assess the effect of tTIS on reinforcement-related benefits in motor learning and the associated neural changes, the participants performed six blocks of 36 trials in the MRI machine, with concurrent tTIS during training, delivered with a Δ*f* of 20 Hz (tTIS_20Hz_) or 80 Hz (tTIS_80Hz_) or as a sham (tTIS_Sham_; 3 tTIS_TYPE_ × 2 Reinf_TYPE_ conditions; Fig. 1b,c). The order of the conditions was balanced among the 24 participants, ensuring that any potential carry-over effect would have the same impact on each experimental condition. To determine the best electrode montage to stimulate the human striatum (putamen, caudate and nucleus accumbens (NAc) bilaterally), computational modelling with a realistic head model was conducted with Sim4Life^59^ (Methods). The selected montage (F3–F4 and TP7–TP8) generated a theoretical temporal interference electric field that was ~30–40% stronger in the striatum than in the overlying cortex, reaching magnitudes of 0.5 to 0.6 V m^−1^ (Fig. 1d,e).

### tTIS_80Hz_ disrupts reinforcement learning of motor skills

**Fig. 2:**
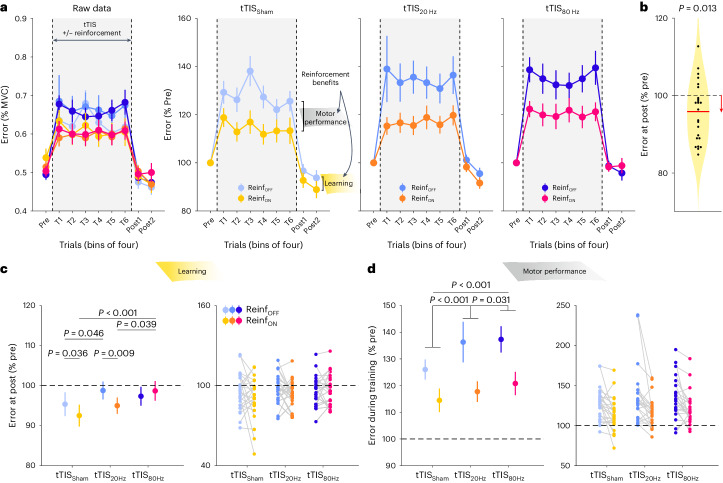
Behavioural results. **a**, Motor performance across training. The raw Error data (expressed in percentage of maximum voluntary contraction (MVC)) from the 24 participants are presented in the left panel for the different experimental conditions in bins of four trials. The increase in Error during training is related to the visual uncertainty (that is, the intermittent disappearance of the cursor) that was applied to enhance reinforcement effects. The three plots on the right represent the pre-training normalized Error in the tTIS_Sham,_ tTIS_20Hz_ and tTIS_80Hz_ blocks. Reinforcement-related benefits represent the improvement in the Error measured in the Reinf_ON_ and Reinf_OFF_ blocks during training (reflecting benefits in motor performance) or at post-training (reflecting benefits in learning). **b**, Averaged learning across conditions. The violin plot shows the Error distribution at post-training (expressed in percentage of pre-training) averaged across conditions, as well as individual participant data. A single-sample two-sided *t*-test showed that the post-training Error was lower than the pre-training level, indicating significant learning in the task (*P* = 0.013; *n* = 24 participants). **c**, Motor learning. The averaged Error at post-training (normalized to pre-training) and the corresponding individual data points in the different experimental conditions are shown in the left and right panels, respectively, for the participants included in the analysis (that is, after outlier detection; remaining *n* = 23). The reduction of Error at post-training reflects true improvement at tracking the target in test conditions (in the absence of reinforcement, visual uncertainty or tTIS). The LMM run on these data revealed a specific effect of tTIS_80Hz_ on reinforcement-related benefits in learning (analysis of variance (ANOVA) with Satterthwaite approximation followed by two-sided pairwise comparisons via estimated marginal means with Tukey adjustment). Learning was disrupted with Reinf_ON_ in the tTIS_80Hz_ condition compared with the tTIS_20Hz_ (*P* = 0.039) and tTIS_Sham_ (*P* < 0.001) conditions. **d**, Motor performance. The averaged Error during training (normalized to pre-training) and the corresponding individual data points in the different experimental conditions are shown in the left and right panels, respectively, for the participants included in the analysis (that is, after outlier detection; *n* = 23). The Error change during training reflects the joint contribution of the experimental manipulations (visual uncertainty, potential reinforcement and tTIS) to motor performance. The LMM run on these data showed a frequency-dependent effect of tTIS on motor performance, irrespective of reinforcement (ANOVA with Satterthwaite approximation followed by two-sided pairwise comparisons via estimated marginal means with Tukey adjustment). Motor performance was disrupted irrespective of reinforcement in the tTIS_20Hz_ (versus tTIS_Sham_: *P* < 0.001) and tTIS_80Hz_ (versus tTIS_Sham_: *P* < 0.001; versus tTIS_20Hz_: *P* = 0.031) conditions. The data are represented as mean ± s.e.

Task performance was evaluated by means of the Error, which was defined as the absolute difference between the applied and target force averaged across samples for each trial, as done previously^5,55,58^ (Fig. 2a). Across conditions, the post-training Error was lower than the pre-training Error (single-sample two-sided *t*-test on the normalized post-training data: *t*_24_ = −2.69; *P* = 0.013; Cohen’s *d* = −0.53; 95% confidence interval (CI), (−0.99, −0.09)), indicating significant motor learning during the task (Fig. 2b). Such improvement was greater when participants had trained with reinforcement (Reinf_TYPE_ effect in the linear mixed model (LMM): *F*_1,1062.2_ = 5.17; *P* = 0.023; partial eta-squared (*η*_p_^2^), 0.005; 95% CI, (0.00, 0.02)), confirming the beneficial effect of reinforcement on motor learning^7,57^. Crucially, though, this effect depended on the type of stimulation applied during training (Reinf_TYPE_ × tTIS_TYPE_ interaction: *F*_2,1063.5_ = 2.11; *P* = 0.034; *η*_p_^2^ = 0.006; 95% CI, (0.00, 0.02); Fig. 2c). While reinforcement significantly improved learning when training was performed with tTIS_Sham_ (two-sided Tukey-corrected pairwise comparison: *P* = 0.036; *d* = −0.22; 95% CI, (−0.46, 0.01)) and tTIS_20Hz_ (*P* = 0.0089; *d* = −0.27; 95% CI, (−0.51, −0.04)), this was not the case with tTIS_80Hz_ (*P* = 0.43; *d* = 0.083; 95% CI, (−0.14, 0.31)). Consistently, direct between-condition comparisons showed that in the Reinf_ON_ condition, learning was reduced with tTIS_80Hz_ compared with tTIS_20Hz_ (*P* = 0.039; *d* = 0.26; 95% CI, (0.02, 0.49)) and tTIS_Sham_ (*P* < 0.001; *d* = 0.45; 95% CI, (0.19, 0.72)), while there was no evidence for a difference between tTIS_20Hz_ and tTIS_Sham_ (*P* = 0.15; *d* = 0.20; 95% CI, (−0.04, 0.43)). This disruption of motor learning with tTIS_80Hz_ was not observed in the absence of reinforcement (tTIS_80Hz_ versus tTIS_20Hz_: *P* = 0.59; *d* = −0.10; 95% CI, (−0.33, 0.12); tTIS_80Hz_ versus tTIS_Sham_: *P* = 0.34; *d* = 0.15; 95% CI, (−0.08, 0.38)). These results point to the fact that high gamma striatal tTIS specifically disrupts the benefits of reinforcement for motor learning and not motor learning in general.

Although training with tTIS_20Hz_ did not alter the benefits of reinforcement for motor learning, we found that learning without reinforcement was significantly impaired in this condition (tTIS_20Hz_ versus tTIS_Sham_: *P* = 0.046; *d* = 0.25; 95% CI, (0.01, 0.49); Fig. 2c). This suggests that tTIS_20Hz_ may disrupt a qualitatively different mechanism involved in motor learning from sensory feedback^60^, in line with the role of striatal beta oscillations in sensorimotor function^28^.

Next, we evaluated the effect of tTIS on motor performance during training itself. As shown in Fig. 2a, the Error was generally higher during training than in test trials due to the presence of visual uncertainty during this phase. The extent of this disruption was reduced in the presence of reinforcement (Reinf_TYPE_: *F*_1,3262.4_ = 339.89; *P* < 0.001; *η*_p_^2^ = 0.09; 95% CI, (0.08, 0.11)), demonstrating the ability of participants to exploit real-time reinforcement information to improve tracking (Fig. 2d). Notably, this effect was not modulated by tTIS_TYPE_ (Reinf_TYPE_ × tTIS_TYPE_: *F*_2,3265.8_ = 0.91; *P* = 0.40; *η*_p_^2^ = 6 × 10^−^^4^), indicating that tTIS did not directly influence reinforcement gains during tracking. However, striatal stimulation did impact general tracking performance independently of reinforcement, as indicated by a significant tTIS_TYPE_ effect (tTIS_TYPE_: *F*_2,3262.4_ = 42.85; *P* < 0.001; *η*_p_^2^ = 0.03; 95% CI, (0.02, 0.04)). This effect was due to an increase in the Error when tTIS_20Hz_ was applied (*P* < 0.001; *d* = 0.28; 95% CI, (0.16, 0.39) when compared with tTIS_Sham_), which was even larger during tTIS_80Hz_ (*P* < 0.001; *d* = 0.38; 95% CI, (0.25, 0.52) and *P* = 0.031; *d* = 0.11; 95% CI, (0.02, 0.20) when compared with tTIS_Sham_ and tTIS_20Hz_, respectively). An additional analysis showed that the detrimental effect of tTIS on motor performance was actually due to an impaired ability to improve performance during training (LMM with continuous fixed effect Trial: tTIS_TYPE_ × Trial interaction: *F*_2,3399_ = 4.46; *P* = 0.012; *η*_p_^2^ = 0.003; 95% CI, (0.00, 0.01); post hoc tests: tTIS_Sham_ versus tTIS_20Hz_: *P* = 0.013; *d* = −0.02; 95% CI, (−0.03, 0.00); tTIS_Sham_ versus tTIS_80Hz_: *P* = 0.068; *d* = −0.01; 95% CI, (−0.03, 0.00); tTIS_20Hz_ versus tTIS_80Hz_: *P* = 0.81; *d* = 0.004; 95% CI, (−0.01, 0.02); Supplementary Fig. 1f). However, again, this effect did not depend on the presence of reinforcement (Reinf_TYPE_ × tTIS_TYPE_ × Trial: *F*_2,3399_ = 0.51; *P* = 0.60; *η*_p_^2^ = 3 × 10^−^^4^). We also found that the detrimental effect of striatal tTIS did not depend on the availability of visual information on the cursor, but rather that tTIS had a general effect on motor performance irrespective of visual and reinforcement feedback (Supplementary Information). This analysis also confirmed that reinforcement gains in motor performance were stronger when visual information was not available (Supplementary Fig. 1g), in line with the behavioural data mentioned above (Supplementary Fig. 1b) and previous studies^56,61^. Overall, these results suggest that striatal tTIS altered motor performance in a frequency-dependent manner but did not influence the ability to rapidly adjust motor commands on the basis of reinforcement feedback during training. Hence, tTIS_80Hz_ may not disrupt real-time processing of reinforcement feedback but may instead impair the beneficial effect of reinforcements on the retention of motor memories^6,7^.

To further understand this dissociation, we ran additional analyses exploring the relationship between reinforcement gains in the training (performed with partial visual feedback and Reinf_ON_ or Reinf_OFF_) and post-training phases (performed with full visual feedback and no reinforcement). We found consistent positive associations between individual reinforcement gains at the end of training (T6) and at the beginning of post-training (Post1) in the tTIS_Sham_ (robust linear regression: *R*^2^ = 0.45, *P* < 0.001) and tTIS_20Hz_ (*R*^2^ = 0.36, *P* = 0.003) conditions and in the additional behavioural dataset (*R*^2^ = 0.31, *P* = 0.009, Supplementary Fig. 2). This association was abolished specifically in the tTIS_80Hz_ condition (*R*^2^ = 0.028, *P* = 0.39): participants who benefited from reinforcement during training did not exhibit gains in learning at post-training (see Supplementary Information for more details on this analysis). This suggests that the disruption of reinforcement motor learning with tTIS_80Hz_ did not concern all participants (in this case, we would still have found a correlation but an upward shift in the intercept) but primarily affected participants who actually benefited from reinforcement during training, further supporting the idea of a specific disruption of reinforcement motor learning with tTIS_80Hz_.

These effects could not be explained by potential differences in initial performance between conditions (Reinf_TYPE_ × tTIS_TYPE_: *F*_2,519.99_ = 1.08; *P* = 0.34; *η*_p_^2^ = 0.004; 95% CI, (0.00, 0.02)), by changes in the flashing properties of the reinforcement feedback (that is, the frequency of colour change during tracking; Reinf_TYPE_ × tTIS_TYPE_: *F*_2,3283_ = 0.19; *P* = 0.82; *η*_p_^2^ = 1 × 10^−^^4^) or by differences in success rate in the Reinf_ON_ blocks (that is, the proportion of success feedback during tracking; tTIS_TYPE_: *F*_2,1702_ = 0.17; *P* = 0.84; *η*_p_^2^ = 2 × 10^−^^4^). There was also no evidence that the Reinf_TYPE_ × tTIS_TYPE_ effect on learning was influenced by the order of the reinforcement conditions (analysis on sub-groups based on whether participants experienced Reinf_ON_ or Reinf_OFF_ first; no Reinf_TYPE_ × tTIS_TYPE_ × Group_TYPE_ interaction: *F*_2,1105.06_ = 1.75; *P* = 0.17; *η*_p_^2^ = 0.003; 95% CI, (0.00, 0.01); see Supplementary Information for more details on these analyses).

Finally, we confirmed that these results were not a consequence of inefficient blinding. During debriefing after the experiment, only 6/24 participants were able to successfully identify the order of the stimulation applied (for example, real–real–placebo; chance level, 4/24; Fisher exact test on proportions, *P* = 0.74). Consistently, the magnitude (Supplementary Fig. 3a) and type (Supplementary Fig. 3b) of tTIS-evoked sensations evaluated before the experiment were qualitatively similar across conditions, and tTIS was generally well tolerated in all participants (no adverse events reported). This suggests that blinding was successful and is unlikely to explain our findings. More generally, this indicates that tTIS evokes very limited sensations (for example, only 2/24 and 1/24 participants rated sensations evoked at 2 mA as “strong” for tTIS_20Hz_ and tTIS_80Hz_, respectively; Supplementary Fig. 3a) that are compatible with efficient blinding.

### Behavioural effect of tTIS_80Hz_ is linked to striatal modulation

As mentioned above, task-based fMRI was acquired during training with concomitant tTIS. This allowed us to evaluate the neural effects of tTIS and their potential relationship to the behavioural effects reported above. As a first qualitative evaluation of the data, we performed a whole-brain analysis in the tTIS_Sham_ condition to assess the network activated during reinforcement motor learning (Reinf_ON_ condition). Consistent with previous neuroimaging studies employing similar tasks^62,63^, we found prominent BOLD activations in a motor network including the putamen, thalamus, cerebellum and sensorimotor cortex, particularly in the left hemisphere, contralateral to the trained hand (Supplementary Fig. 4 and Supplementary Table 1). However, contrasting Reinf_ON_ and Reinf_OFF_ conditions did not reveal any significant cluster at the whole-brain level. This first analysis thus did not reveal any region specifically activated in the presence of reinforcement, but rather confirms the involvement of a motor network engaged in this type of task irrespective of the reinforcement feedback.

**Fig. 3:**
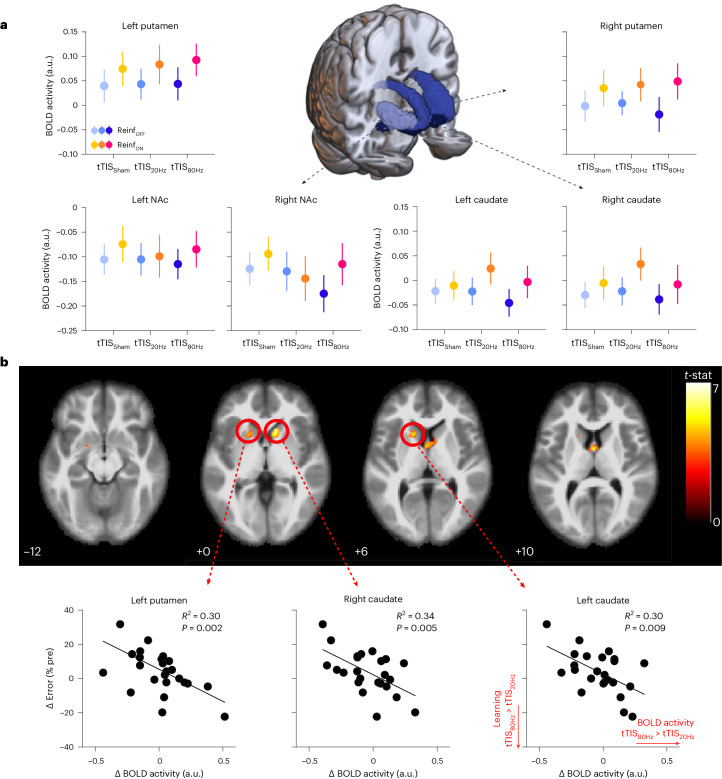
Striatal activity. **a**, Striatal BOLD responses. A 3D reconstruction of the striatal masks used in the current experiment is surrounded by plots showing averaged BOLD activity for each mask in the different experimental conditions. An LMM run on these data showed higher striatal responses in the Reinf_ON_ than in the Reinf_OFF_ condition, but no effect of tTIS_TYPE_ and no interaction (*n* = 24 participants). The data are represented as mean ± s.e. **b**, Whole-brain activity associated with the behavioural effect of tTIS_80Hz_ on reinforcement motor learning. The correlation between tTIS-related modulation of striatal activity (tTIS_80Hz_–tTIS_20Hz_) and learning abilities in the Reinf_ON_ condition (*n* = 24) is shown. Significant clusters of correlation were found in the left putamen and bilateral caudate (*t*-contrast; uncorrected *P* = 0.001 at the voxel level; corrected cluster-based false discovery rate, *P* = 0.05). The lower panel shows individual robust linear regressions for the three significant regions highlighted in the whole-brain analysis.

As a second step, we evaluated the effect of tTIS on striatal activity, as a function of the type of reinforcement feedback and focusing on the same regions of interest (ROIs) that were used to optimize tTIS exposure in the modelling. We extracted averaged BOLD activity within the bilateral putamen, caudate and NAc based on the Brainnetome Atlas (BNA)^64^ in the different experimental conditions and considered these six striatal ROIs (ROI_STR_) as fixed effects in the LMM. This model revealed a significant enhancement of striatal activity with Reinf_ON_ with respect to Reinf_OFF_ (*F*_1,800.01_ = 13.23; *P* < 0.001; *η*_p_^2^ = 0.02; 95% CI, (0.00, 0.04)), consistent with previous literature^11^, but no tTIS_TYPE_ effect (*F*_2,800.01_ = 0.46; *P* = 0.63; *η*_p_^2^ = 0.001; 95% CI, (0.00, 0.01)) and no interaction (all *P* > 0.65; Fig. 3a). Despite the absence of effects of tTIS on averaged striatal activity, we then asked whether the behavioural effects of tTIS_80Hz_ on reinforcement motor learning (that is, tTIS_80Hz_ versus tTIS_20Hz_ and tTIS_Sham_ with Reinf_ON_) could be linked to the modulation of activity in core brain regions. To do so, we ran a whole-brain analysis focusing on the main behavioural effects mentioned above. The results revealed that the effect of tTIS_80Hz_ (with respect to tTIS_20Hz_) on motor learning in the Reinf_ON_ condition was specifically related to the modulation of activity in two clusters encompassing the left putamen and bilateral caudate (Fig. 3b and Supplementary Table 2). The presence of the high-frequency carrier (kHz) in both stimulation conditions rules out the possibility that the correlation was due to putative neuromodulatory effects of high-frequency stimulation. No significant clusters were found for the tTIS_80Hz_–tTIS_Sham_ contrast or for the control tTIS_20Hz_–tTIS_Sham_ contrast, indicating that the reported correlation is not due to a general link between striatal activity and reinforcement motor learning. Overall, these results provide evidence that the detrimental effect of tTIS_80Hz_ on reinforcement learning of motor skills is related to the modulation of neural activity specifically in the striatum.

### tTIS_80Hz_ enhances striatum-to-frontal-cortex connectivity

**Fig. 4:**
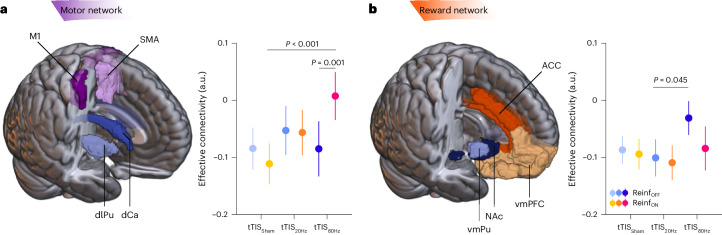
Striatum-to-frontal-cortex effective connectivity. **a**, Motor network. A 3D reconstruction of the masks used for the motor network (that is, dorso-lateral putamen (dlPu), dorsal caudate (dCa), M1 and SMA) is shown on the left. The plot on the right shows the effective connectivity from motor striatum to motor cortex in the different experimental conditions (*n* = 24 participants). Note the increase of connectivity with tTIS_80Hz_ in the presence of reinforcement (tTIS_80Hz_–Reinf_ON_: *P* = 0.001 (versus tTIS_80Hz_–Reinf_OFF_) and *P* < 0.001 (versus tTIS_Sham_–Reinf_ON_)). **b**, Reward network. A 3D reconstruction of the masks used for the reward network (that is, ventro-medial putamen (vmPu), NAc, vmPFC and ACC) is shown on the left. The plot on the right shows the effective connectivity from motor striatum to motor cortex in the different experimental conditions (*n* = 24). ROIs were defined on the basis of the BNA^12^. In **a** and **b**, the outputs of LMMs were analysed using ANOVA with Satterthwaite approximation followed by two-sided pairwise comparisons via estimated marginal means with Tukey adjustment. The data are represented as mean ± s.e.

Interactions between the striatum and frontal cortex are crucial for a variety of behaviours, including motor and reinforcement learning^13^. In particular, reinforcement motor learning requires the use of information about task success to guide future motor commands^5^, a process in which the striatum may play an integrative role at the interface between fronto-striatal loops involved in reward processing and motor control^13,65^. In a subsequent analysis, we asked whether striatal tTIS modulates striatum-to-frontal-cortex communication during reinforcement motor learning. More specifically, we computed effective connectivity (using the generalized psychophysiological interactions (gPPI) method^66^) between striatal and frontal regions classically associated with motor and reward-related functions, and thought to be involved in reinforcement motor learning^67,68^. For the motor network, we evaluated effective connectivity between motor parts of the striatum (that is, dorso-lateral putamen and dorsal caudate) and two regions strongly implicated in motor learning: the medial part of the supplementary motor area (SMA) and the part of the primary motor cortex (M1) associated with upper limb functions (Fig. 4a). For the reward network, we assessed connectivity between parts of the striatum classically associated with limbic functions (that is, the NAc, the ventro-medial putamen and two frontal areas involved in reward processing: the anterior cingulate cortex (ACC) and the ventro-medial prefrontal cortex (vmPFC); Fig. 4b)^11^. The LMM run with the fixed effects Reinf_TYPE_, tTIS_TYPE_ and Network_TYPE_ showed a significant effect of tTIS_TYPE_ (*F*_2,2264.0_ = 5.42; *P* = 0.005; *η*_p_^2^ = 0.005; 95% CI, (0.00, 0.01)) that was due to higher connectivity in the tTIS_80Hz_ condition than in tTIS_Sham_ (Tukey-corrected *P* = 0.004; *d* = 0.16; 95% CI, (0.05, 0.28)). There was no significant difference in connectivity between tTIS_80Hz_ and tTIS_20Hz_ (*P* = 0.069; *d* = 0.11; 95% CI, (0.00, 0.22)) or between tTIS_20Hz_ and tTIS_Sham_ (*P* = 0.58; *d* = 0.051; 95% CI, (−0.05, 0.16)). Hence, tTIS_80Hz_, but not tTIS_20Hz_, enhanced effective connectivity between the striatum and frontal cortex during motor training. This increase in effective connectivity with tTIS_80Hz_ actually led to a connectivity closer to the resting state (values closer to 0; Methods). Put differently, while the task induced a reduction in effective connectivity between striatum and frontal cortex, tTIS_80Hz_ disrupted this modulation by bringing connectivity back to the resting state.

The LMM did not reveal any effect of Reinf_TYPE_ (*F*_1,2264.0_ = 0.010; *P* = 0.92; *η*_p_^2^ = 5 × 10^−6^), Network_TYPE_ (*F*_1,2264.0_ = 3.16; *P* = 0.076; *η*_p_^2^ = 0.001; 95% CI, (0.00, 0.01)) or a double interaction (Reinf_TYPE_ × Network_TYPE_: *F*_1,2264.0_ = 3.52; *P* = 0.061; *η*_p_^2^ = 0.002; 95% CI, (0.00, 0.01)). Yet, we did find a significant Reinf_TYPE_ × tTIS_TYPE_ × Network_TYPE_ interaction (*F*_2,2264.0_ = 4.87; *P* = 0.008; *η*_p_^2^ = 0.004; 95% CI, (0.00, 0.01)). This triple interaction was related to the fact that tTIS_80Hz_ increased connectivity in the Reinf_ON_ condition in the motor network (Reinf_ON_ versus Reinf_OFF_: *P* = 0.001; *d* = 0.33; 95% CI, (0.11, 0.55); Fig. 4a), while this effect was not observed in the reward network (*P* = 0.063; *d* = −0.19; 95% CI, (−0.40, 0.02); Fig. 4b). There was no evidence for such an increase in either of the two networks when either tTIS_Sham_ or tTIS_20Hz_ was applied (all *P* > 0.40, all *d* [−0.09, −0.02]). Moreover, in the motor network, connectivity in the Reinf_ON_ condition was higher with tTIS_80Hz_ than with tTIS_Sham_ (*P* < 0.001; *d* = 0.42; 95% CI, (0.19, 0.65)). This effect did not reach significance when contrasting tTIS_80Hz_ with tTIS_20Hz_ (*P* = 0.059; *d* = 0.23; 95% CI, (0.02, 0.44); Fig. 4a). These data suggest that tTIS_80Hz_ enhanced the neuromodulatory influence of the striatum on motor cortex during task performance, but only in the presence of reinforcement. In the reward network, post hoc tests revealed that connectivity in the Reinf_OFF_ condition was significantly higher with tTIS_80Hz_ than with tTIS_20Hz_ (*P* = 0.045; *d* = 0.24; 95% CI, (0.03, 0.46); Fig. 4b), in line with the general effect of tTIS_TYPE_ on connectivity reported above. This pattern of results suggests that the increase of connectivity from striatum to frontal cortex observed with tTIS_80Hz_ depends on the presence of reinforcement, in particular in the motor network. This reinforcement-dependent increase of connectivity may reflect the preferential effect of tTIS_80Hz_ on striatal gamma oscillations^69^ in a situation where these oscillations are already boosted by the presence of reinforcement^19^ (Discussion).

In a subsequent analysis, we verified that these results did not depend on the specific frontal ROIs considered in the analysis (ROI_TYPE_: M1 and SMA in the motor network and ACC and vmPFC in the reward network). Importantly, we did not find a tTIS_TYPE_ × Reinf_TYPE_ × ROI_TYPE_ interaction in the motor network (*F*_2,1112_ = 0.83; *P* = 0.44; *η*_p_^2^ = 0.001; 95% CI, (0.00, 0.01)) or in the reward network (*F*_2,1112_ = 0.61; *P* = 0.54; *η*_p_^2^ = 0.001; 95% CI, (0.00, 0.01)), suggesting that the main connectivity results were consistent within a network and were not influenced by the specific frontal ROI included in the analysis (see Supplementary Information for more details on this analysis). As an additional control, we verified that the effects of tTIS_TYPE_ on connectivity could not be observed in a control network associated with language (as defined by ref. ^70^), which was unlikely to be involved in the present task and did not include the striatum (Methods). As expected, there was no evidence for a modulation of effective connectivity within the language network by Reinf_TYPE_ (*F*_1,547_ = 0.81; *P* = 0.37; *η*_p_^2^ = 0.001; 95% CI, (0.00, 0.01)) or by tTIS_TYPE_ (*F*_2,547_ = 0.58; *P* = 0.56; *η*_p_^2^ = 0.002; 95% CI, (0.00, 0.01)) or by Reinf_TYPE_ × tTIS_TYPE_ (*F*_2,547_ = 0.45; *P* = 0.64; *η*_p_^2^ = 0.002; 95% CI, (0.00, 0.01)). Hence, tTIS-related and reinforcement-related changes in connectivity were consistent within the considered fronto-striatal networks and not observed in a control network unrelated to the task.

Contrary to the BOLD results presented above, we did not find any correlations between the effects of tTIS_80Hz_ on connectivity and motor learning, in either the motor network (robust linear regression: tTIS_80Hz_–tTIS_Sham_: *R*^2^ = 0.019, *P* = 0.48; tTIS_80Hz_–tTIS_20Hz_: *R*^2^ = 0.034, *P* = 0.54) or the reward network (tTIS_80Hz_–tTIS_Sham_: *R*^2^ = 0.037, *P* = 0.46; tTIS_80Hz_–tTIS_20Hz_: *R*^2^ < 0.001, *P* = 0.75), suggesting some degree of independence between the effect of tTIS_80Hz_ on reinforcement motor learning and that on effective connectivity.

Overall, these results highlight the ability of tTIS_80Hz_, but not tTIS_20Hz_, to modulate striatum-to-frontal-cortex connectivity, depending on the presence of reinforcement. However, the absence of a correlation with behaviour suggests that this effect may not be directly associated with the detrimental effect of tTIS_80Hz_ on reinforcement motor learning or that tTIS_80Hz_-related changes in striato-frontal communication were linked to other aspects of reinforcement learning not captured by our task.

### Neural effects of tTIS_80Hz_ depend on impulsivity

Determining individual factors that shape responsiveness to non-invasive brain stimulation approaches is a crucial step towards better understanding the mechanisms of action as well as envisioning the stratification of patients in future clinical interventions^71^. A potential factor that could explain inter-individual differences in responsiveness to tTIS_80Hz_ is the level of impulsivity. Impulsivity has been associated with changes of gamma oscillatory activity in the striatum of rats^72^ and with the activity of fast-spiking interneurons in the striatum^73,74^, a neuronal population that is strongly entrained to gamma rhythms^19,21^ and may therefore be particularly sensitive to tTIS_80Hz_. In a subsequent exploratory analysis, we asked whether the neural effects of tTIS_80Hz_ were associated with impulsivity levels, as evaluated by a well-established independent delay-discounting questionnaire performed at the beginning of the experiment^75,76^. A whole-brain analysis revealed that impulsivity was associated with the effect of tTIS_80Hz_ on BOLD activity (with respect to tTIS_20Hz_) specifically in the left caudate nucleus (Supplementary Fig. 5a,b and Supplementary Table 3). Moreover, the effect of tTIS_80Hz_ on striatum-to-motor-cortex connectivity reported above was negatively correlated with impulsivity when contrasting tTIS_80Hz_ with both tTIS_Sham_ (Supplementary Fig. 5c, left) and tTIS_20Hz_ (Supplementary Fig. 5c, middle). Such correlations were absent when contrasting tTIS_20Hz_ with tTIS_Sham_ (Supplementary Fig. 5c, right), as well as when considering the same contrasts in the reward instead of the motor network (see Supplementary Information for more details). Taken together, these results suggest that inter-individual variability in impulsivity might influence the neural responses to striatal tTIS_80Hz_.

## Discussion

In this study, we combined striatal tTIS with electric field modelling, behavioural and fMRI analyses to evaluate the causal role of the striatum in reinforcement learning of motor skills in healthy humans. tTIS_80Hz_, but not tTIS_20Hz_, disrupted the ability to learn from reinforcement feedback. This behavioural effect was associated with modulation of neural activity specifically in the striatum. We also show that tTIS_80Hz_, but not tTIS_20Hz_, increased the neuromodulatory influence of the striatum on connected frontal cortical areas involved in reinforcement motor learning. Finally, inter-individual variability in the neural effects of tTIS_80Hz_ could be partially explained by impulsivity, suggesting that this trait may constitute a determinant of responsiveness to high gamma striatal tTIS. Overall, the present study shows that striatal tTIS can non-invasively modulate a striatal mechanism involved in reinforcement learning, expanding our tools for the study of causal relationships between deep brain structures and human behaviour.

We investigated the causal role of the human striatum in reinforcement learning of motor skills in healthy humans, a question that cannot be addressed with conventional non-invasive brain stimulation techniques. In particular, by stimulating at different frequencies, we aimed to dissociate striatal mechanisms involved in reinforcement and sensorimotor learning. In line with our main hypothesis, we found that striatal tTIS_80Hz_ altered reinforcement learning of a motor skill. Such disruption was frequency- and reinforcement-specific: learning was not altered with striatal tTIS_20Hz_ in the presence of reinforcement, or when striatal tTIS_80Hz_ was delivered in the absence of reinforcement. The rationale to stimulate at high gamma frequency was based on previous work showing reinforcement-related modulation of gamma oscillations in the striatum^19–21,24,26,72,77^ and in the frontal cortex^77–80^. Several neuronal mechanisms may contribute to the detrimental effect of tTIS_80Hz_ on reinforcement motor learning. First, as tTIS_80Hz_ consisted of a constant high gamma oscillating field applied on the striatum, it may have perturbed the encoding of reinforcement information into high gamma oscillations^19–21,25–27^, preventing participants from learning the motor skill on the basis of different outcomes. Put differently, tTIS_80Hz_ may specifically saturate high gamma activity in the striatum, preventing reinforcement-related modulations^81^. Moreover, because reinforcement motor learning probably engages synchronized activity in a network of regions including fronto-striatal loops, neuromodulation of a single node of the circuit may alter the synchronization of activity in the network^81^ and the temporal coordination with interacting rhythms^25^. Finally, because we did not have access to electrophysiological recordings of oscillatory activity in the striatum, the applied stimulation was not personalized, as it did not take into account the individual high gamma frequency peak associated with reward processing and the potential heterogeneity of gamma activity within the striatum^24^. Hence, tTIS_80Hz_ may have resulted in a frequency mismatch between the endogenous high gamma activity and the externally imposed rhythm, which could paradoxically result in a reduction of neuronal entrainment, in particular when the frequency mismatch is relatively low^82^. Importantly, in contrast to striatal tTIS_80Hz_, we found that tTIS_20Hz_ reduced learning, but only in the absence of reinforcement. This result fits well with the literature linking striatal beta oscillations to sensorimotor functions^28,29,31,83–85^. Taken together, an interpretation of these results is that different oscillations in the striatum support qualitatively distinct motor learning mechanisms, with beta activity contributing mostly to sensory-based learning and high gamma activity being particularly important for reinforcement learning. This being said, it is important to note that because we do not have concurrent electrophysiological recordings in the striatum, we cannot be sure whether the effects of tTIS_20Hz_ and tTIS_80Hz_ were related to frequency-specific interactions with beta or high gamma rhythms, respectively, or rather resulted from different broadband responses when stimulating at these frequencies. Yet, these results still suggest that sensory- and reinforcement-based motor learning rely on partially different neural mechanisms, in line with previous literature^8,9,60,68,86,87^.

Striatal tTIS also impaired tracking performance during training, irrespective of the presence of reinforcement. This frequency-dependent reduction of motor performance may be due to altered neuronal processing in the sensorimotor striatum that may lead to less fine-tuned motor control abilities^88^. Importantly, though, tTIS did not modulate the ability of participants to benefit from real-time reinforcement feedback during motor performance. This suggests that striatal tTIS_80Hz_ altered the beneficial effects of reinforcement on learning (as evaluated in test conditions at post-training), but not on motor performance (as evaluated during training). This dissociation between the effects of striatal tTIS_80Hz_ on reinforcement-related gains in motor performance and in learning may be explained by the fact that these two phases of the protocol probe different processes^7,55,58,89–91^. While the improvement of motor performance with reinforcement relies on rapid feedback corrections based on expected outcomes^67,92–96^, reinforcement gains in learning (that is, probed in test conditions without reinforcement) may rather reflect the beneficial effect of reinforcement on the retention of motor memories^4,7,55,90^. This idea that the mechanisms underlying performance changes in training and retention phases are partially different is well supported by previous motor learning literature^6,8,97^. For instance, in sensorimotor adaptation paradigms, the presence of reward boosts motor memory retention but not the adaptation process itself^7,86,90,91,98,99^, and M1 transcranial direct current stimulation modulates the effect of reward on retention but has no effect on the training phase^90^. Such dissociation also appears to generalize to other motor learning tasks^18,100^, including force-tracking paradigms^55^ (see also Supplementary Fig. 1b). Importantly, while reinforcement gains in motor performance and learning seem to reflect the operation of partially dissociable mechanisms, it is no surprise that these processes are correlated at the group level (Supplementary Fig. 2), as they may be influenced by common individual factors (for example, sensitivity to reward)^101^. In contrast, the absence of correlation in the striatal tTIS_80Hz_ condition suggests that the stimulation particularly impaired reinforcement gains in learning in the participants who initially benefited from reinforcement during training (Supplementary Fig. 2a). Hence, a potential explanation for the present results is that striatal tTIS_80Hz_ did not disrupt rapid motor corrections based on recent outcomes during training, but may rather have altered the strengthening of the memory trace based on reinforcements^6,7^. Overall, these results are compatible with the view that specific patterns of oscillatory activity in the striatum are involved in motor control and learning processes^31^ and can be modulated with electrical stimulation^69,102,103^.

To better understand the neural effects and frequency specificity of tTIS, we coupled striatal tTIS and task performance with simultaneous fMRI acquisition. The imaging results support the view that the effect of tTIS_80Hz_ on reinforcement learning of motor skills was indeed related to neuromodulation of the striatum. When considering averaged BOLD activity, we found a general increase of striatal activity when reinforcement was provided^11^, but no effect of tTIS. Crucially, though, the detrimental effect of tTIS_80Hz_ on reinforcement learning was related to a specific modulation of activity in the caudate and putamen, providing evidence that the present behavioural effects were indeed driven by focal neuromodulation of the striatum (Fig. 3). Interestingly, participants with stronger disruption of reinforcement learning at the behavioural level were also the ones exhibiting stronger suppression of striatal activity with tTIS_80Hz_ (than with tTIS_20Hz_), suggesting that tTIS-induced reduction of striatal activity is detrimental for reinforcement motor learning. Further analyses showed that tTIS_80Hz_, but not tTIS_20Hz_, increased the neuromodulatory influence of the striatum on frontal areas known to be important for motor learning and reinforcement processing^97,104^. More specifically, tTIS_80Hz_ disrupted the task-related decrease in connectivity observed with tTIS_Sham_ and tTIS_20Hz_, bringing connectivity closer to resting-state values. This effect depended on the type of network considered (reward versus motor) and on the presence of reinforcement. Striatal tTIS_80Hz_ coupled with reinforcement increased connectivity between the motor striatum and the motor cortex, while this effect was not observed when considering the connectivity between limbic parts of the striatum and prefrontal areas involved in reward processing (Fig. 4). This result may reflect the differential influence of striatal tTIS on distinct subparts of the striatum, depending on their pattern of activity during the task^53^. A recent study in non-human primates showed that transcranial alternating current stimulation can have opposite effects on neuronal activity depending on the initial entrainment of neurons to the target frequency^82^. Hence, the present differential effects of tTIS_80Hz_ on motor and reward striato-frontal pathways may be due to different initial patterns of activity in these networks in the presence of reinforcement. Electrophysiological recordings with higher temporal resolution than fMRI are required to confirm or infirm this hypothesis. Overall, the present neuroimaging results support the idea that the behavioural effects of striatal tTIS_80Hz_ on reinforcement learning are associated with a selective modulation of striatal activity that influences striato-frontal communication.

The fact that we observed increased connectivity with tTIS_80Hz_ and at the same time a disruption of behaviour may appear contradictory at first glance. Yet, multiple lines of evidence indicate that increases in connectivity are not necessarily beneficial for behaviour. For instance, the severity of motor symptoms in Parkinson’s disease is associated with excessive connectivity in the beta band, and the reduction of such connectivity with treatment is associated with clinical improvement^29,32^. Moreover, there is evidence that excessive functional^105,106^ as well as structural^107,108^ connectivity in fronto-striatal circuits is associated with impulsivity. Hence, the increase in connectivity observed with tTIS_80Hz_ appears to be compatible with the behavioural findings. This being said, contrary to the BOLD results, we did not find any correlation between the effects of tTIS_80Hz_ on connectivity and on reinforcement motor learning, suggesting some degree of independence between these two effects. Future studies could aim at determining whether tTIS_80Hz_-related changes in striato-frontal communication are linked to other aspects of reward processing that are not captured by our reinforcement motor learning task.

From a methodological point of view, the present results provide experimental support for the idea that the effects of tTIS are related to amplitude modulation of electric fields deep in the brain and not to the high-frequency fields themselves, in line with recent work^41,42,52,53^. The different behavioural and neural effects of striatal tTIS_80Hz_ and tTIS_20Hz_ despite comparable carrier frequencies (centred on 2 kHz) indicate that temporal interference was indeed the driving force of the present effects. Moreover, the disruption of reinforcement motor learning with tTIS_80Hz_ (relative to tTIS_20Hz_) was specifically related to neuromodulation of the striatum, where the amplitude of the tTIS field was highest according to our simulations (see refs. ^51,52^ for recent validations of comparable simulations in cadaver experiments). Hence, we believe that the frequency- and reinforcement-dependent tTIS effects reported here cannot be explained by direct modulation of neural activity by the high-frequency fields. Yet, disentangling the neural effects of the low-frequency envelope and the high-frequency carrier appears to be an important next step to better characterize the mechanisms underlying tTIS^47^. We also note that the tTIS field strengths achieved according to our simulations (in the range of 0.5–0.6 V m^−1^) were sufficient to induce behavioural and neural effects, in line with recent data^52,53^ (see also ref. ^48^). Determining the minimum effective dose for tTIS is an important line of future research given recent simulation results suggesting that stimulation via amplitude modulation with high-frequency carrier signals (such as those arising during tTIS) may require higher dosages than conventional electrical stimulation with low frequencies (such as during transcranial alternating current stimulation), probably due to the low-pass-filtering properties of neurons^43,109^.

Finally, the strength of the behavioural effects of tTIS can be considered small to medium^110^ (*d* = 0.2–0.5). We note that these effect sizes are consistent with studies applying other types of non-invasive brain stimulation in healthy young adults, in the context of both motor learning (see ref. ^111^ for a meta-analysis) and reward tasks (for example, refs. ^112,113^), despite the much longer stimulation time used in these studies (between 3 and 20 times longer). Moreover, when expressed relative to the plateau of performance in the task (Supplementary Fig. 1d), the effect of tTIS_80Hz_ represents a complete disruption of a ~24% reinforcement-related learning gain (Supplementary Fig. 1e). Overall, although they are moderate, we believe that the present effect sizes are relevant and consistent with what can be expected from the non-invasive brain stimulation literature.

### Limitations

The present study includes some limitations that we would like to acknowledge. First, at the imaging level, we did not find a significant effect of reinforcement at the whole-brain level. This might be due to the short duration of the task (6 × 40 s), combined with the fact that we did not couple reinforcement to monetary incentives, a manipulation known to boost striatal activity in the context of motor learning^18^. Yet, when considering BOLD activity in the striatal ROIs, we did find a significant effect of reinforcement, suggesting that our experimental manipulation did increase striatal activity but that the strength of the effect was insufficient to survive at the whole-brain level. Second, we did not find any effect of tTIS when considering averaged BOLD activity. Again, the short duration of the blocks may contribute to this non-significant effect. Another possible interpretation is that the effect of tTIS on BOLD activity is not uniform across participants, as it probably depends on individual anatomy and function of the targeted brain region, as observed for other non-invasive brain stimulation techniques^114^. Consistently, we found a correlation between levels of impulsivity and the neural effects of tTIS_80Hz_ (both BOLD and connectivity; Supplementary Fig. 5). Importantly, though, when including learning as a behavioural regressor, we did find significant clusters of correlation specifically in the striatum (Fig. 3), suggesting that the behavioural effects were indeed related to modulation of activity in the target region. This result was significant when contrasting tTIS_80Hz_ to the active control (tTIS_20Hz_), but not to tTIS_Sham_. Overall, we believe that the fMRI data do support the idea that the behavioural effects of the stimulation were indeed related to modulation of neural activity in the striatum, also in line with the present simulations on realistic head models (Fig. 1) and the connectivity results (Fig. 4). This idea is also in agreement with another recent study investigating the effects of tTIS on motor sequence learning^53^. However, a limitation of the present dataset is the very short duration of stimulation and imaging for each experimental condition, which may explain some inconsistencies in the results. Hence, following this proof-of-concept study showing robust behavioural effects and related neural changes, future studies including longer fMRI and stimulation sessions are required to further confirm these results.

Finally, in the present study, the computational modelling was performed on a realistic, detailed head model (that is, the MIDA model^59^; Methods). One limitation of this approach is that the electric field simulations do not take individual structural information into account. Such individual modelling would require information on brain anisotropy, an aspect that is likely to significantly influence tTIS exposure^44,115^. However, in the present study, diffusion MRI to evaluate fractional anisotropy was not acquired. Future studies including diffusion MRI data will allow for personalized modelling, paving the way for individualized tTIS informed by brain structure^52^.

## Conclusion

The present findings show the ability of non-invasive striatal tTIS to interfere with reinforcement learning in humans through selective modulation of striatal activity and support the causal functional role of the human striatum in reinforcement motor learning. This deep brain stimulation was well tolerated and compatible with efficient blinding, suggesting that tTIS provides the option to circumvent the steep depth–focality trade-off of current non-invasive brain stimulation approaches in a safe and effective way. Overall, tTIS opens possibilities for the study of causal brain–behaviour relationships and for the treatment of neuropsychiatric disorders associated with alterations of deep brain structures.

## Methods

### Participants

All participants gave their written informed consent in accordance with the Declaration of Helsinki and with the approval of the Cantonal Ethics Committee Vaud, Switzerland (project number 2020-00127). A total of 48 right-handed healthy volunteers participated in the study. Of these, 24 participants were enrolled for the main tTIS study (15 women, 25.3 ± 0.7 years old (mean ± s.e.)). Another group of 24 volunteers participated in the behavioural control experiment (Supplementary Fig. 1b–d; 14 women, 24.2 ± 0.5 years old). Handedness was determined via a shortened version of the Edinburgh Handedness Inventory^116^ (laterality index, 89.3 ± 2.14% for the main study and 86.4 ± 2.51% for the control experiment). None of the participants had any neurological or psychiatric disorder or were taking any centrally acting medication (see Supplementary Information for a complete list of exclusion criteria). Finally, all participants were asked to fill out a delay-discounting monetary choice questionnaire^117^, which evaluates the propensity of participants to choose smaller, sooner rewards over larger, later rewards, a preference commonly associated with choice impulsivity^75,118^. The participants were financially compensated at a standard rate of 20 CHF per hour.

### Experimental procedures

The study employed a randomized, double-blind, sham-controlled design. Following screening and inclusion, the participants were invited to a single experimental session including the performance of a motor learning task with concurrent tTIS of the striatum and fMRI. Overall, the participants practised six blocks of trials, which resulted from the combination of two reinforcement feedback conditions (Reinf_TYPE_: Reinf_ON_ or Reinf_OFF_) with three types of striatal stimulation (tTIS_TYPE_: tTIS_Sham_, tTIS_20Hz_ or tTIS_80Hz_).

#### Motor learning task

##### General aspects

The participants practised an adaptation of a widely used force-tracking motor task^54,55^ with an fMRI-compatible fibre-optic grip-force sensor (Current Designs) positioned in their right hand. This task has the advantage of evaluating learning in a context in which movements have to be dynamically adjusted in response to constantly evolving sensory information. Such careful, continuous force control represents a situation that is relevant in many daily-life activities, including situations involving limited visual feedback such as when learning to drive or to manipulate fragile objects^119^. In these situations, the learner has to use somatosensory information in combination with information about task success to improve future motor commands, a process that might be particularly relevant in tasks where visual information is limited as well as in early stages of motor learning, when the desired sensory state is unknown^5,58,68,120^. In addition to these elements, force-modulation tasks are relevant for rehabilitation as they can be used to evaluate motor function in clinical populations^121,122^.

The task was developed in MATLAB 2018 (MathWorks) exploiting the Psychophysics Toolbox extensions^123,124^ and was displayed on a computer screen with a refresh rate of 60 Hz. The task required the participants to squeeze the force sensor to control a cursor displayed on the screen. Increasing the exerted force resulted in the cursor moving vertically and upward in a linear way. Each trial started with a preparatory period in which a sidebar appeared at the bottom of the screen (Fig. 1a). After a variable time interval (0.9 to 1.1 s), a cursor (a black circle) popped up in the sidebar, and simultaneously a target (a grey larger circle with a cross in the middle) appeared, indicating the start of the movement period. The participants were asked to modulate the force applied on the transducer to keep the cursor as close as possible to the centre of the target. The target moved in a sequential way along a single vertical axis for 7 s. The maximum force required (that is, the force required to reach the target when it was in the uppermost part of the screen; MaxTarget_Force_) was set at 4% of MVC evaluated at the beginning of the experiment. This low force level was chosen on the basis of pilot experiments to limit muscular fatigue. Finally, each trial ended with a blank screen displayed for 2 s before the beginning of the next trial.

##### Trial types and reinforcement manipulation

During the experiment, the participants were exposed to different types of trials (Fig. 1a and Supplementary Video 1). In test trials, the cursor remained on the screen, and the target was consistently displayed in grey for the whole duration of the trial. These trials served to evaluate pre- and post-training performance for each block, without any disturbance. In Reinf_ON_ and Reinf_OFF_ trials (used during training only), we provided only partial visual feedback to the participants to increase the impact of reinforcement on learning^5,56–58^. The cursor was only intermittently displayed during the trial: it was always displayed in the first second of the trial and then disappeared for a total of 4.5 s randomly split in the remaining time by bits of 0.5 s. The cursor was therefore displayed 35.7% of the time during these trials (2.5 s over the 7 s trial). Importantly, unlike the cursor, the target always remained on the screen for the whole trial, and the participants were instructed to continue to track the target even when the cursor was away.

In addition to this visual manipulation, in Reinf_ON_ trials, the participants also trained with reinforcement feedback indicating success or failure of the tracking in real time. The participants were informed that, during these trials, the colour of the target would vary as a function of their performance: the target was displayed in green when tracking was considered successful and in red when it was considered a failure. Online success on the task was determined on the basis of the Error, defined as the absolute force difference between the force required to be in the centre of the target and the exerted force^5,54,55,58^. The Error, expressed as a percentage of MVC, was computed for each frame refresh and allowed to classify a sample as successful or not on the basis of a closed-loop reinforcement schedule^8^. More specifically, for each training trial, a force sample (recorded at 60 Hz, corresponding to the refresh rate of the monitor) was considered successful if the computed Error was below the median Error over the four previous trials at this specific sample. Put differently, to be successful, the participants had to constantly beat their previous performance. This closed-loop reinforcement schedule allowed us to deliver consistent reinforcement feedback across individuals and conditions (see the control analysis on success rates in the Supplementary Information), while maximizing uncertainty on the presence of reinforcement, an aspect that is crucial for efficient reinforcement motor learning^125^. In addition to this closed-loop design, samples were also considered successful if the cursor was very close to the centre of the target (that is, within one radius around the centre, corresponding to an Error below 0.2% of MVC). This was done to prevent any conflict between visual information (provided by the position of the cursor relative to the target) and reinforcement feedback (provided by the colour of the target), which could occur in situations of extremely good performance (when the closed-loop Error cut-off is below 0.2% of MVC).

As a control, Reinf_OFF_ trials were similar to Reinf_ON_ trials, with the only difference being that the displayed colours were either cyan or magenta and were generated randomly. The participants were explicitly told that, in this condition, the colours were displayed randomly and could be ignored. The visual properties of the target in the Reinf_OFF_ condition were designed to match the Reinf_ON_ condition in terms of relative luminance (cyan (RGB, (127.5, 242.1, 255)) matched to green (127.5, 255, 127.5) and magenta (211.7, 127.5, 255) to red (255, 127.5, 127.5)) and average frequency of change in colours (that is, the average number of changes in colours divided by the total duration of a trial; Supplementary Information).

In this task, training trials differed from test trials regarding not only the colour of the target (red/green or cyan/magenta in training trials and grey in test trials) but also the visual feedback experienced (partial and full visual feedback in training and test trials, respectively). This choice was motivated by several reasons. First, we wanted to evaluate learning in the classical, unperturbed, version of the force-tracking task^54,55^, which is compatible with clinical translation. Second, on the basis of additional behavioural data on another group of participants (*n* = 24; Supplementary Fig. 1b–d), we found that significant effects of reinforcement on learning were observed only when training was performed with partial visual feedback (displayed for 35.7% of the trial time, as in the present study), in line with previous results^56,61^. However, this additional study also revealed very limited improvement of performance during training with partial visual feedback, potentially due to ceiling effects on performance in this condition. Yet, the improvement of performance when comparing the pre- and post-training assessments suggested that practising the task with partial visual feedback still induced significant learning of the skill. Finally, the change in visual feedback between training and post-training was the same in all experimental conditions; this aspect of the task is therefore unlikely to explain the reinforcement as well as the stimulation effects reported here.

Even though our study focused on reinforcement motor learning, it is worth mentioning that other learning mechanisms such as error-based or strategic processes are likely to be also engaged during the force-tracking task and may have recruited other brain regions beyond the striatum^4^. Notably, though, our protocol was specifically designed to compare learning in the Reinf_ON_ and Reinf_OFF_ conditions in the same individuals while keeping the other parameters of the task constant, to specifically isolate the contribution of reinforcement processes in motor learning.

##### Motor learning protocol

After receiving standardized instructions about the force-tracking task, the participants practised five blocks of familiarization (total of 75 trials) without tTIS. The first block of familiarization included 20 trials with the target moving in a regular fashion (0.5 Hz sinuoid). Then, in a second block of familiarization, the participants performed 35 trials of practice with an irregular pattern, with the same properties as the training patterns (see below). Finally, we introduced the reinforcement manipulation and let the participants perform two short blocks (eight trials each) including Reinf_ON_ and Reinf_OFF_ trials. These first four blocks of familiarization were performed outside the MRI environment. A final familiarization block (four trials) was performed after installation in the scanner, to allow the participants to get used to performing the task in the MRI machine. This long familiarization allowed the participants to get acquainted with the use of the force sensor before the beginning of the experiment.

During the main part of the experiment, the participants performed six blocks of trials in the MRI machine with concurrent striatal tTIS (Fig. 1b). Each block was composed of 4 pre-training trials followed by 24 training and 8 post-training trials. Pre- and post-training trials were performed in test conditions, without tTIS, and were used to evaluate motor learning. Training trials were performed with or without reinforcement feedback and with concomitant striatal tTIS and were used as a proxy of motor performance. During training, trials were interspersed with 25 s resting periods every four trials (used for fMRI contrasts; see below). The order of the six experimental conditions was pseudo-randomized across participants: the six blocks were divided into three pairs of blocks with the same tTIS condition, and each pair was then composed of one Reinf_ON_ and one Reinf_OFF_ block. Within this structure, the order of the tTIS_TYPE_ and Reinf_TYPE_ conditions were balanced among the 24 participants. Hence, this randomization allowed us to ensure that any order effect that may arise from the repetition of the learning blocks would have the same impact on each experimental condition (for example, four participants experienced tTIS_80Hz_–Reinf_ON_ in the first block, four other participants in the second block, four in the third block and so on).

As mentioned above, the protocol involved multiple evaluations of motor learning within the same experimental session. To limit carry-over effects from one block to the next, each experimental block was associated with a different pattern of movement of the target (Supplementary Fig. 1a). Put differently, in each block, the participants had to generate a new pattern of force to successfully track the target. To balance the patterns’ difficulty, they all consisted of the summation of five sinusoids of variable frequency (range, 0.1–1.5 Hz) that presented the following properties: the average force was between 45% and 55% of MaxTarget_Force_, the absolute average derivative was between 54% and 66% of MaxTarget_Force_ per second and the number of peaks was 14 (defined as an absolute change of force of at least 1% of MaxTarget_Force_). These parameters were determined on the basis of pilot experiments to obtain a relevant level of difficulty for young healthy adults and consistent learning across the different patterns.

#### Striatal tTIS

##### General concept

tTIS is an innovative non-invasive brain stimulation approach, in which two or more independent stimulation channels deliver high-frequency currents in the kHz range (oscillating at *f*_1_ and *f*_1_ + Δ*f*; Fig. 1c). These high-frequency currents are assumed to be too high to effectively modulate neuronal activity^41,50,126^. Still, by applying a small shift in frequency, they result in a modulated electric field with the envelope oscillating at the low-frequency Δ*f* (target frequency) where the two currents overlap. The peak of the modulated envelope amplitude can be steered towards specific areas located deep in the brain, by tuning the positions of the electrodes and the current ratio across stimulation channels^41^ (Fig. 1c,d). On the basis of these properties, tTIS has been shown to be able to focally target the activity of deep structures in rodents, without engaging overlying tissues^41^. Here we applied tTIS via surface electrodes applying a low-intensity, sub-threshold protocol following the currently accepted cut-offs and safety guidelines for low-intensity transcranial electric stimulation in humans^127^.

##### Stimulators

The currents for tTIS were delivered by two independent DS5 isolated bipolar constant current stimulators (Digitimer Ltd). The stimulation patterns were generated using a custom-based MATLAB graphical user interface and transmitted to the current sources using a standard digital–analogue converter (DAQ USB-6216, National Instruments). Finally, an audio transformer was added between stimulators and participants to avoid possible direct current accumulation.

##### Stimulation protocols

During the six training blocks, we applied three different types of striatal tTIS (two blocks each): a stimulation with a tTIS envelope modulated at 20 Hz (tTIS_20Hz_), a stimulation with a tTIS envelope modulated at 80 Hz (tTIS_80Hz_) and a sham stimulation (tTIS_Sham_). For tTIS_20Hz_, the posterior stimulation channel (TP7–TP8; see below) delivered a 1.99 kHz stimulation, while the anterior one delivered a 2.01 kHz stimulation (Δ*f* = 20 Hz). For tTIS_80Hz_, the posterior and anterior channels delivered 1.96 kHz and 2.04 kHz, respectively (Δ*f* = 80 Hz). Hence, in both conditions, the high-frequency component was comparable, and the only difference was Δ*f*. During each block, tTIS was applied for five minutes (6 × 50 s) during training. Each stimulation period started and ended with currents ramping up and down, respectively, for 5 s. tTIS was applied only while the participants were performing the motor task and not during resting periods or pre- and post-training assessments. Finally, tTIS_Sham_ consisted of a ramping-up (5 s) immediately followed by a ramping-down (5 s) of 2 kHz currents delivered without any shift in frequency. This condition allowed us to mimic the sensations experienced during the active conditions tTIS_20Hz_ and tTIS_80Hz_, while delivering minimal brain stimulation (Supplementary Fig. 3). A trigger was sent 5 s before the beginning of each trial to align the beginning of the task and the beginning of the frequency shift after the ramp-up. Other tTIS parameters were set as follows: current intensity per stimulation channel, 2 mA (baseline-to-peak); electrode type, round, conductive rubber with conductive cream/paste; electrode size, 3 cm^2^ (see the ContES checklist in the Supplementary Information for more details).

The stimulation was applied in the MRI environment (Siemens 3T MAGNETOM Prisma; Siemens Healthcare) using a standard RF filter module and MRI-compatible cables (neuroConn GmbH). The technological, safety and noise tests and the methodological factors can be found in the Supplementary Information (Supplementary Table 4) and are based on the ContES checklist^128^.

##### Modelling

Electromagnetic simulations were carried out to identify optimized electrode placement and current steering parameters. The simulations were performed using the MIDA head model^59^, a detailed anatomical head model featuring >100 distinguished tissues and regions that was derived from multi-modal image data of a healthy female volunteer. Importantly, for brain stimulation modelling, the model differentiates different scalp layers, skull layers, grey and white matter, cerebrospinal fluid, and the dura and accounts for electrical conductivity anisotropy and neural orientation on the basis of diffusion tensor imaging data. Circular electrodes (radius, 0.7 cm) were positioned on the skin according to the 10–10 system, and the electromagnetic exposure was computed using the ohmic-current-dominated electro-quasistatic solver from Sim4Life v.5.0 (ZMT Zurich MedTech AG), which is suitable due to the dominance of ohmic currents over displacement currents and the long wavelength compared with the simulation domain^129^. Dielectric properties were assigned on the basis of the IT’IS Tissue Properties Database v.4.0 (ref. ^130^). Rectilinear discretization was performed, and grid convergence as well as solver convergence analyses were used to ensure negligible numerical uncertainty, resulting in a grid that included more than 54 million voxels. Dirichlet voltage boundary conditions and then current normalization were applied. The electrode–head interface contact was treated as ideal. tTIS exposure was quantified according to the maximum modulation envelope magnitude formula from Grossman et al.^41^. A sweep over 960 permutations of the four electrode positions was then performed, considering symmetric and asymmetric montages with parallel (sagittal and coronal) or crossing current paths, while quantifying bilateral striatum (putamen (BNA regions 225, 226, 229 and 230), caudate (BNA regions 219, 220, 227 and 228) and NAc (BNA regions 223 and 224)) exposure performance according to three metrics: (1) target exposure strength, (2) focality ratio (the ratio of target tissue volume above the threshold compared to the whole-brain tissue volume above the threshold, a measure of stimulation selectivity) and (3) activation ratio (the percentage of target volume above the threshold with respect to the total target volume, a measure of target coverage). We defined the threshold as the 98th volumetric iso-percentile level of the tTIS. From the resulting Pareto-optimal front, two configurations stood out particularly: one that maximized focality and activation (AF3–AF4 and P7–P8) and one that accepted a reduction of these two metrics by a quarter, while increasing the target exposure strength by more than 50% (F3–F4 and TP7–TP8). This last montage was selected to ensure sufficient tTIS exposure in the striatum^53^ (Fig. 1c,d).

##### Electrode positioning and stimulation-related sensations

On the basis of the modelling approach described above, we defined the stimulation electrode positions in the framework of the EEG 10–10 system^131^. The optimal montage leading in terms of target (that is, bilateral striatum) exposure strength and selectivity was composed of the following electrodes: F3, F4, TP7 and TP8. Their locations were marked with a pen on the scalp, and, after skin preparation (cleaning with alcohol), round conductive rubber electrodes of 3 cm^2^ were placed, adding a conductive paste (Ten20, Weaver; or Abralyt HiCl, Easycap GmbH) as an interface to the skin. The electrodes were held in position with tape, and the cables were oriented towards the top to allow good positioning inside the scanner. Impedances were checked and optimized until they were below 20 kΩ (ref. ^48^). Once good contact was obtained, we tested different intensities of stimulation for each stimulation protocol to familiarize the participants with the perceived sensations and to systematically document them. tTIS_Sham_, tTIS_20Hz_ and tTIS_80Hz_ were applied for 20 seconds with the following increasing current amplitudes per channel: 0.5 mA, 1 mA, 1.5 mA and 2 mA. The participants were asked to report any kind of sensation, and, if a sensation was felt, they were asked to grade the intensity from 1 to 3 (light to strong) as well as give at least one adjective to describe it (Supplementary Fig. 3). Following this step, the cables were removed and replaced by MRI-compatible cables, and a bandage was added to apply pressure on the electrodes and keep them in place. An impedance check was repeated in the MRI machine right before the training and then again at the end of all recordings.

#### MRI data acquisition

Structural and functional images were acquired using a 3T MAGNETOM PRISMA scanner (Siemens). T1-weighted images were acquired via the 3D MPRAGE sequence with the following parameters: repetition time (TR), 2.3 s; echo time (TE), 2.96 ms; flip angle, 9°; slices, 192; voxel size, 1 × 1 × 1 mm^3^; field-of-view (FOV), 256 mm. Anatomical T2 images were also acquired with the following parameters: TR, 3 s; TE, 409 ms; flip angle, 120°; slices, 208; voxel size, 0.8 × 0.8 × 0.8 mm^3^; FOV, 320 mm. Finally, functional images were recorded using echo-planar imaging sequences with the following parameters: TR, 1.25 s; TE, 32 ms; flip angle, 58°; slices, 75; voxel size, 2 × 2 × 2 mm^3^; FOV, 112 mm.

### Data and statistical analyses

Data and statistical analyses were carried out with MATLAB 2018a (MathWorks) and the R software environment (version 2021) for statistical computing and graphics^132^. Robust linear regressions were fitted with the MATLAB function robustfit. LMMs were fitted using the lmer function of the lme4 package in R^133^. As random effects, we added intercepts for participants and block. The normality of residuals and the homoscedasticity of the data were systematically checked, and logarithmic transformations were applied when necessary (that is, when the skewness of the residuals’ distribution was not between −2 and 2 (ref. ^134^) or when homoscedasticity was violated on the basis of visual inspection). To mitigate the impact of isolated influential data points on the outcome of the final model, we used tools of the influence.ME package to detect and remove influential cases on the basis of the following criterion: distance > 4 × mean distance^135^. Statistical significance was determined using the anova function with Satterthwaite’s approximations of the lmerTest package^136^. For specific post hoc comparisons, we conducted two-sided pairwise tests by computing estimated marginal means with the emmeans package with Tukey adjustment of *P* values to correct for multiple comparisons^137^. Standardized effect size measures were obtained using the eff_size function of the emmeans package^138^ and the eta_squared function of the effectsize package^139^. The level of significance was set at *P* < 0.05.

#### Behavioural data

##### Evaluation of motor learning

The main goal of the present study was to evaluate the influence of striatal tTIS on reinforcement motor learning. To do so, we first removed trials in which participants did not react within 1 s after the appearance of the cursor and target, considering that these extremely long preparation times may reflect substantial fluctuations in attention^140^. This occurred extremely rarely (0.52% of the whole dataset). For each participant and each trial, we then quantified the tracking Error as the absolute force difference between the applied and required force, as done previously^5,55,58^. Tracking performance during training and post-training trials was then normalized according to each participant’s initial level by expressing the Error data as a percentage of the average pre-training Error for each block. To test our main hypothesis predicting specific effects of striatal tTIS on reinforcement motor learning, we performed an LMM on the post-training data with tTIS_TYPE_ and Reinf_TYPE_ as fixed effects. We then ran the same analysis on the training data, to evaluate whether striatal tTIS also impacted motor performance while stimulation was being delivered.

As a control, we checked that initial performance at pre-training was not different between conditions with an LMM on the Error data obtained at pre-training. Again, tTIS_TYPE_ and Reinf_TYPE_ were considered as fixed effects. Finally, another LMM was fitted with the fixed effect tTIS_TYPE_ to verify that the amount of positive reinforcement (as indicated by a green target) in the Reinf_ON_ blocks was similar across tTIS_TYPES_.

#### fMRI data

##### Imaging preprocessing

We analysed the functional imaging data using Statistical Parametric Mapping v.12 (Wellcome Department of Cognitive Neurology) implemented in MATLAB R2018a (MathWorks). All functional images underwent a common preprocessing procedure including the following steps: slice time correction, spatial realignment to the first image, normalization to the standard MNI space and smoothing with a 6 mm full-width half-maximal Gaussian kernel. T1 anatomical images were then co-registered to the mean functional image and segmented. This allowed us to obtain bias-corrected grey and white matter images by normalizing the functional images via the forward deformation field. To select participants with acceptable levels of head movement, framewise displacement was calculated for each run. A visual check of both non-normalized and normalized images was performed to ensure good preprocessing quality. Finally, possible tTIS-related artefacts were investigated on the basis of signal-to-noise ratio maps (see below).

##### Signal-to-noise ratio

Total signal-to-noise ratio maps were computed to check the presence of possible artefacts induced by the electrical stimulation. The values were calculated for each voxel by dividing the mean of the voxel time series by its standard deviation. Spherical ROIs were then defined both underneath the tTIS electrodes and at four different locations distant from the electrodes as a control. The centre of each spherical ROI was obtained by projecting the standard MNI coordinates of each electrode on the scalp^141^ towards the centre of the brain. After visual inspection of the ROIs, average total signal-to-noise ratio maps were extracted within each sphere. An LMM was used to compare the average signal-to-noise ratio underneath the electrodes versus the control regions and between stimulation protocols. The results of this analysis are presented in the Supplementary Information (Supplementary Fig. 6).

##### Task-based BOLD activity analysis

A general linear model was implemented at the single-participant level to estimate signal amplitude. Eight regressors were included in the model: six head motion parameters (displacement and rotation) and normalized time series within the white matter and the cerebrospinal fluid. Linear contrasts were then computed to estimate specific activity during the motor task with respect to resting periods. Functional activation was also extracted within specific ROIs individually defined on the basis of structural images. More specifically, the Freesurfer recon-all function was run on the basis of the structural T1w and T2w images (Freesurfer v.7.1.1, https://surfer.nmr.mgh.harvard.edu/; coded in Bash (v.4.4.20(1)-release) and Python (v.3.8.3)). The BNA parcellation was derived on the individual participant space, and the selected ROIs were then co-registered to the functional images and normalized to the MNI space. BOLD activity within the individual striatal masks was averaged and compared between different striatal nuclei—namely, the putamen (BNA regions 225, 226, 229 and 230), caudate (BNA regions 219, 220, 227 and 228) and NAc (BNA regions 223 and 224). Comparisons between conditions were presented for uncorrected *P* = 0.001 at the voxel level and multiple comparison corrected at the cluster level to reduce the false discovery rate, *P* = 0.05.

##### Effective connectivity analyses

As an additional investigation, we computed task-modulated effective functional connectivity by means of the CONN toolbox 2021a (www.nitrc.org/projects/conn, RRID:SCR_009550) running in MATLAB R2018a (MathWorks). An additional denoising step was added by applying band-pass filtering from 0.01 to 0.1 Hz and by regressing potential confounders (white matter, cerebrospinal fluid and realignment parameters). After that, gPPI connectivity was extracted within specific pre-defined customized sub-networks: a reward network and a motor network. gPPI evaluates condition-specific changes in effective connectivity, defined as the directed effect that one brain region has on another under some model of neuronal coupling^142^. In particular, gPPI considers a series of equations in which activity in a ROI (pre-defined frontal areas in our case) depends on a specific condition (the ‘psychological’ factor) and on activity in the seed region (striatum here, the ‘physiological’ factor). By solving these equations, it is possible to determine a coefficient that represents task modulation of effective connectivity^143^. Importantly, task-related changes in effective connectivity are expressed relative to rest, and therefore values closer to 0 reflect a connectivity similar to the resting state.

The reward network was defined as follows: two regions within the striatum, the NAc (BNA regions 223 and 224) and the ventro-medial putamen (BNA regions 225 for left and 226 for right), and two frontal areas, the ACC (BNA regions 177, 179 and 183 for left and 178, 180 and 184 for right) and the orbitofrontal cortex within the vmPFC (BNA regions 41, 45, 47, 49 and 187 for left and 42, 46, 48, 50 and 188 for right). The motor network included the following areas: the dorso-lateral putamen (BNA 229 for left and 230 for right), the dorsal caudate (BNA regions 227 for left and 228 for right), the medial part of the SMA (BNA regions 9 for left and 10 for right) and the part of the M1 associated with upper limb function (BNA regions 57 for left and 58 for right). We considered connectivity in the left and right motor and reward networks regardless of laterality. These ROIs were selected on the basis of the following rationale. First, they are consistent with previous literature on reinforcement learning of motor skills^68,90,144,145^. Second, there is structural and functional evidence for these fronto-striatal connections^146,147^. Third, the frontal areas included in the analyses are well-established hubs of the motor learning (M1 and SMA; see ref. ^12^ for a meta-analysis) and reward networks (vmPFC and ACC; see ref. ^11^ for a meta-analysis). Finally, gPPI was also extracted within a control language network, defined on the basis of the functional atlas described by Shirer et al.^70^.

### Reporting summary

Further information on research design is available in the Nature Portfolio Reporting Summary linked to this article.

### Supplementary information

Supplementary Information Supplementary Figs. 1–6 and Tables 1–4.

Reporting Summary

Supplementary Video 1 Example trials of the motor learning task in the Reinf_ON_ condition. Note that only 4 trials of training are shown here (compared with 24 trials per block in the experiment) and 4 trials of post-training (compared with 8 trials per block in the experiment). Here the video was shot outside the MRI machine, similarly as during familiarization.

## Data Availability

All data necessary to generate the main results and figures are available in the Zenodo repository (10.5281/zenodo.10458885)^148^. The BNA was used and can be downloaded from http://atlas.brainnetome.org/. The tissue properties used for the modelling of electric fields were based on the IT’IS Tissue Properties Database v.4.0 and can be downloaded here: https://itis.swiss/virtual-population/tissue-properties/overview/.

## Code availability

The scripts necessary to generate the main results are available in the Zenodo repository (10.5281/zenodo.10458885)^148^.
