# Supplementary material for: Non-invasive stimulation of the human striatum disrupts reinforcement learning of motor skills
Source: Nat Hum Behav. 2024 May 29;8(8):1581–98. doi: 10.1038/s41562-024-01901-z (PMC11343719; doi:10.1038/s41562-024-01901-z)
Supplement: Supplementary file 1 — Supplementary Figs. 1–6 and Tables 1–4. [file 41562_2024_1901_MOESM1_ESM.pdf]

nature human behaviour

Supplementary information

https://doi.org/10.1038/s41562-024-01901-z

Non-invasive stimulation of the human striatum disrupts reinforcement learning of motor skills

In the format provided by the authors and unedited

Supplementary information

Non-invasive stimulation of the human striatum disrupts reinforcement learning of motor skills

Pierre Vassiliadis, Elena Beanato, Traian Popa, Fabienne Windel, Takuya Morishita, Esra Neufeld, Julie Duque, Gerard Derosiere, Maximilian J. Wessel and Friedhelm C. Hummel

1. Additional behavioural experiment

To determine the optimal experimental parameters to study reinforcement learning of motor skills, we performed an additional behavioural experiment, in the absence of brain stimulation and imaging. In particular, we tested the relationship between the amount of visual feedback available during Training and the benefits of reinforcement in the force-tracking task. Another group of young healthy participants (n=24; 14 women, 24.2 ± 0.5 years old, independent from the subjects tested in the main experiment) performed blocks of the task with $Reinf_{ON}$ or $Reinf_{OFF}$ and with either full visual feedback or only partial visual feedback (cursor displayed for 35.7% of the total trial duration, as in the main study). Each learning block was composed of 30 trials (vs. 36 trials in the main study) and in addition to real-time closed-loop reinforcement feedback, participants also received endpoint feedback on their overall performance after each trial during Training (i.e., indicating success or failure on the trial). The LMM ran on the Post-training data revealed a significant effect of visual feedback ($F_{(1,788.33)}$=5.90; p=0.015, η_p^2=0.007, 95% CI [0.00, 0.02]), reinforcement ($F_{(1,787.87)}$=11.64; p<0.001, η_p^2=0.01, 95% CI [0.00, 0.04]) and a significant interaction between these two factors ($F_{(1,788.03)}$=10.27; p=0.001, η_p^2=0.01, 95% CI [0.00, 0.03], **Figure S1b**). Interestingly, Tukey-corrected post-hoc tests showed that the interaction was due to the fact that while there was no evidence for an improvement of learning with reinforcement when Training was performed with full visual feedback (p=0.88, d=-0.014, 95% CI [-0.23, 0.20] for the $Reinf_{ON}$-$Reinf_{OFF}$ contrast), reinforcement induced robust benefits when training with partial visual feedback (p<0.001, d=-0.46, 95% CI [0.72, 0.21] **Figure S1c**). This result is in line with previous literature

showing that reinforcement feedback is particularly beneficial for motor learning when visual feedback is uncertain[1,2]. Based on the outcome of this additional study, we decided to train participants with partial visual feedback in the present experiment to evaluate the effect of tTIS in a version of the task that yielded significant reinforcement gains. Notably, this work also shows that the effect of reinforcement on motor learning observed in the $tTIS_{Sham}$ and $tTIS_{20Hz}$ conditions (Figure 2) is reproducible.

2. Plateau of performance in the task.

In the same independent cohort of participants (see above, Figure S1b, c), we also evaluated tracking performance changes following extensive training on a single sequence (150 trials, >6 times more training than in the training phase of the main study). We found that on average, participants were able to reduce the Error by 18.7% after extensive practice on this task (**Figure S1d**). In the light of these data, the reinforcement gains reported in the additional behavioural experiment and in the $tTIS_{Sham}$ condition of the main study represent ~24.6% and ~24.2% of this maximal performance improvement, respectively (**Figure S1e**). Put differently, the presence of reinforcement during the 24 trials of Training brings participants 24% closer to the maximum possible level of performance achievable in this task (when trained extensively towards the plateau of improvement). Notably, $tTIS_{80Hz}$, but not $tTIS_{20Hz}$, completely disrupted these learning gains (Figure S1e).

3. Evolution of motor performance in the different conditions

The main analysis revealed a general effect of tTIS on motor performance during Training, irrespective of the presence of reinforcement. As a subsequent analysis, we also asked whether the evolution of performance during Training depended on type of striatal stimulation applied. We ran the same LMM as in the main study (see Results) but with the addition of a continuous fixed effect Trial, allowing us to evaluate whether the slope of performance change was different according to $tTIS_{TYPE}$. (**Figure S1f**). Indeed, this analysis

revealed a significant $tTIS_{TYPE}$ x Trial interaction ($F_{(2, 3399)}$=4.46; p=0.012, η_p^2=0.003, 95% CI [0.00, 0.01]) that was due a different slope with $tTIS_{Sham}$ compared to $tTIS_{20Hz}$ (p=0.013 d=-0.02, 95% CI [-0.03, 0.00]). Evolution of performance was not significantly different when contrasting $tTIS_{80Hz}$ to $tTIS_{Sham}$ (p=0.068, d=-0.01, 95% CI [0.00, 0.03]) and $tTIS_{20Hz}$ (p=0.81, d=-0.004, 95% CI [-0.02, 0.00]). Notably, this effect could not be explained by differences in initial performance (all p>0.21 when comparing intercepts). Moreover, there was no evidence for a different effect of tTIS on motor improvement during Training depending on the presence of reinforcement ($Reinf_{TYPE}$ x $tTIS_{TYPE}$ x Trial: $F_{(2, 3399)}$=0.51; p=0.60, η_p^2=3x10^{-4}). Overall, this analysis shows that the detrimental effect of striatal tTIS on motor performance is due to an impaired ability to improve performance with practice and further confirms that tTIS did not modulate the ability to use reinforcement feedback during Training.

4. Effect of visual and reinforcement feedback on motor performance

As a control, we asked whether the tTIS and reinforcement effects reported in Figure 2 depended on the availability of visual information during Training. To do so, we computed the normalised Error for phases with the $Cursor_{ON}$ or $Cursor_{OFF}$ (taking into account a lag of 0.25s, corresponding to the estimated visuo-motor delay in this type of task for young healthy subjects[3]) and analysed these data in a LMM including the factors $Reinf_{TYPE}$, $tTIS_{TYPE}$ and $Cursor_{TYPE}$. As in the main analysis, we confirmed the effect of $Reinf_{TYPE}$ ($F_{(1, 6872)}$=344.87; p<0.001, η_p^2=0.05, 95% CI [0.04, 0.06]), $tTIS_{TYPE}$ ($F_{(2,6872)}$=28.79; p<0.001, η_p^2=0.008, 95% CI [0.00, 0.01]) and the absence of interaction between these two factors ($F_{(2,6875.4)}$=0.49; p=0.61, η_p^2=1x10^{-4}, **Figure S1g**). This analysis also revealed a $Cursor_{TYPE}$ effect ($F_{(2,6875.3)}$=49.66; p<0.001, η_p^2=0.007, 95% CI [0.00, 0.01]) which was due to the fact that the Error was generally higher in the absence visual information on the position of the cursor (d=0.17, 95% CI [0.10, 0.24] for the $Cursor_{OFF}$-$Cursor_{ON}$ contrast). Interestingly, there was also a $Reinf_{TYPE}$ x $Cursor_{TYPE}$ interaction ($F_{(2,6872)}$=29.35; p<0.001, η_p^2=0.004, 95% CI [0.00, 0.01]): while benefits of reinforcement were significant in both the $Cursor_{ON}$ (p<0.001, d=-0.32, 95% CI [-0.43, -0.20])

and $Cursor_{OFF}$ ($p<0.001$, $d=-0.58$, 95% CI [-0.76, -0.40]) conditions, the magnitude of the reinforcement-related gains in performance were larger in the $Cursor_{OFF}$ condition (two-sided t-test comparing the Error gains in $Cursor_{OFF}$ and $Cursor_{ON}$: $t_{(46)}=2.74$, $p=0.009$, $d=-0.78$, 95% CI [-1.36, -0.18]). Moreover, post-hoc tests also revealed that the absence of vision of the cursor was detrimental for performance in the $Reinf_{OFF}$ condition ($p<0.001$, $d=0.30$, 95% CI [0.19, 0.41]) but not in presence of $Reinf_{ON}$ ($p=0.25$, $d=0.04$, 95% CI [-0.03, 0.11]). Hence, the presence of reinforcement was particularly beneficial when visual information was not available, in line with previous research[1,2] and also in agreement with the results of our additional experiment (Figure S1b, c). Importantly, the LMM did not reveal any interaction between $tTIS_{TYPE}$ and $Cursor_{TYPE}$ ($F_{(2,6872)}=0.49$; $p=0.31$, $\eta_p^2=3x10^{-4}$) and no triple interaction ($F_{(2,6872)}=1.53$; $p=0.22$, $\eta_p^2=4x10^{-4}$), confirming that striatal tTIS had a global effect on motor performance during Training, which did not seem to depend on the presence of visual and reinforcement feedback.

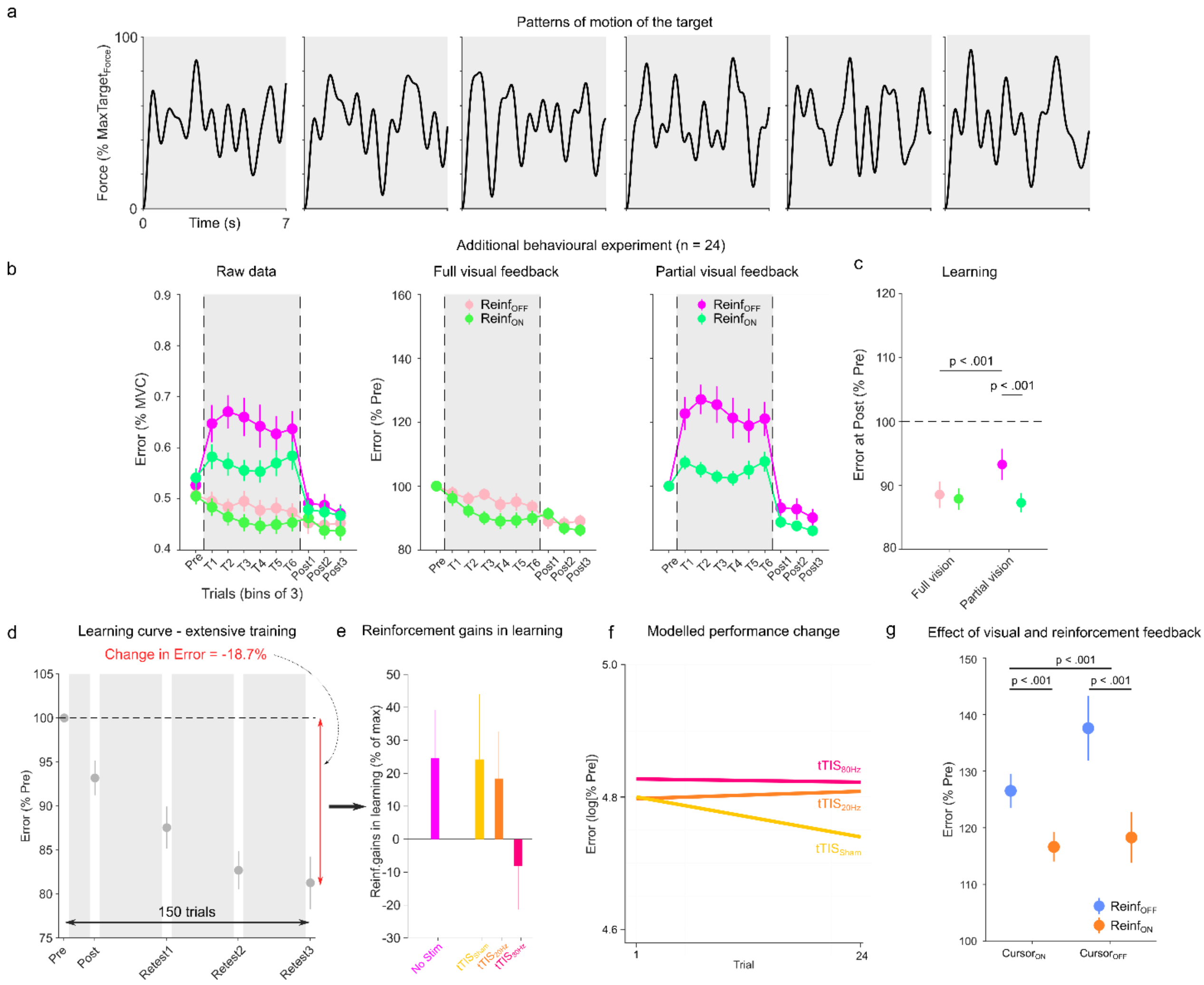

Figure S1. Supplementary information and analyses on the behaviour. a) Patterns of motion of the target. For each block of training, participants had to learn a new pattern of motion of the target. The patterns had similar mathematical properties and their relationship to a condition was randomised (see Methods for more details). **b, c) Results of an additional behavioural experiment (n = 24). b) Motor performance across training.** Raw Error data (expressed in % of Maximum Voluntary Contraction [MVC]) are presented on the left panel for the different experimental conditions in bins of 3 trials. On the right, the two plots represent the Pre-training normalised Error in the Full visual feedback and Partial visual feedback blocks (i.e., cursor displayed 35.7% of the time, as in the main experiment). Note the substantial gains in motor performance, especially with partial visual feedback but also the limited improvement of performance during training in this condition. **c) Motor learning.** Averaged Error at Post-training (normalised to Pre-training) in the different experimental conditions are shown, for the subjects included in the analysis (i.e., after outlier detection, n=23). Reduction of Error at Post-training reflects true improvement at tracking the target in Test conditions (in the absence of reinforcement or visual uncertainty). The LMM ran on these data revealed significant a significant effect of reinforcement feedback on learning when training with partial (two-sided Tukey-corrected post-hoc test: $p<0.001$), but not full ($p=0.88$), visual feedback. **d) Learning curve after extensive practice on the task on an independent dataset** (n=24, same participants as in b and c). The average reduction of Error after 150 trials of training was 18.74% of the Pre-training performance. **e) Reinforcement gains in learning expressed with respect to maximum learning estimated on the task.** Learning gains were similar in the additional experiment, in the absence of stimulation and in the $tTIS_{Sham}$ and $tTIS_{20Hz}$ conditions. Only $tTIS_{80Hz}$ disrupted this effect. **f) Slopes of performance change during Training in the different stimulation conditions.** Modeled performance change for

$tTIS_{Sham}$, $tTIS_{20Hz}$ and $tTIS_{80Hz}$ throughout Training. The $tTIS_{TYPE}$ x Trial interaction revealed that performance improved more with $tTIS_{Sham}$ compared to $tTIS_{20Hz}$ (two-sided Tukey-corrected post-hoc test: p=0.013). Notably this effect was not modulated by the presence of reinforcement and could not be explained by differences in intercepts. **g) Effect of visual and reinforcement feedback on motor performance.** Pre-training normalised Error depending on the presence of the cursor ($Cursor_{ON}$ or $Cursor_{OFF}$), and the presence of reinforcement feedback during Training (n = 24 subjects). The significant $Reinf_{TYPE}$ x $Cursor_{TYPE}$ interaction (p<0.001) was related to the fact that the benefits of reinforcement were stronger when visual information was not available (two-sided t-test comparing the gains: p=0.009). Notably, this analysis takes into account a visuo-motor delay of 0.25s, as previously reported during a similar task (Lam and Zenon, 2021). Outputs of LMMs were analysed using ANOVA with Satterthwaite approximation followed by two-sided pairwise comparisons via estimated marginal means with Tukey adjustment, in the case of significant effects in the ANOVA. Data are represented as mean ± SE.

5. Dissociation between tTIS effects at Training and Post-training stages

To further explore the relationship between tTIS effects on reinforcement gains on motor performance and learning, we conducted an additional analysis focusing on the transition between the Training and Post-training assessments (T6 to Post1). We evaluated how much inter-individual variability in reinforcement gains in the end of Training (i.e., at T6, performed with partial visual feedback and $Reinf_{ON}$ or $Reinf_{OFF}$) was associated with individual improvements in the beginning of the Post-training stage (i.e., Post1, performed with full visual feedback and no reinforcement). As shown on **FigureS2a**, we found a significant positive association between individual reinforcement gains in the end of training (T6) and in the beginning of Post-training (Post1) in the $tTIS_{Sham}$ condition (robust linear regression: R^2=0.45, p<0.001). Participants who were more responsive to reinforcement during Training were also the ones showing stronger effects at Post-training. Notably, this correlation was replicated within the same participants in the $tTIS_{20Hz}$ condition (R^2=0.36, p=0.003), and also in the additional behavioural experiment presented in Figure S1b. Interestingly, in this case the correlation was observed not only when training was performed with partial visual feedback (as in the main study, R^2=0.31, p=0.009), but also in the full visual feedback condition (R^2=0.26, p=0.013, **Figure S2b**), a situation in which we found significant reinforcement gains in Training but not at Post-training (as with $tTIS_{80Hz}$). Crucially, this association was abolished specifically in the $tTIS_{80Hz}$ condition (R^2=0.028, p=0.39): participants who benefited from reinforcement

during Training did not exhibit gains in performance at Post-training. This suggests that the disruption of Post-training reinforcement gains with $tTIS_{80Hz}$ did not concern all participants (in this case we would have still found a correlation but an upward shift in the intercept), but primarily affected participants who actually benefited from reinforcement during Training. This analysis further supports the idea of a specific disruption of reinforcement motor learning with $tTIS_{80Hz}$ which was not observed in the 4 control conditions taken from 2 independent datasets.

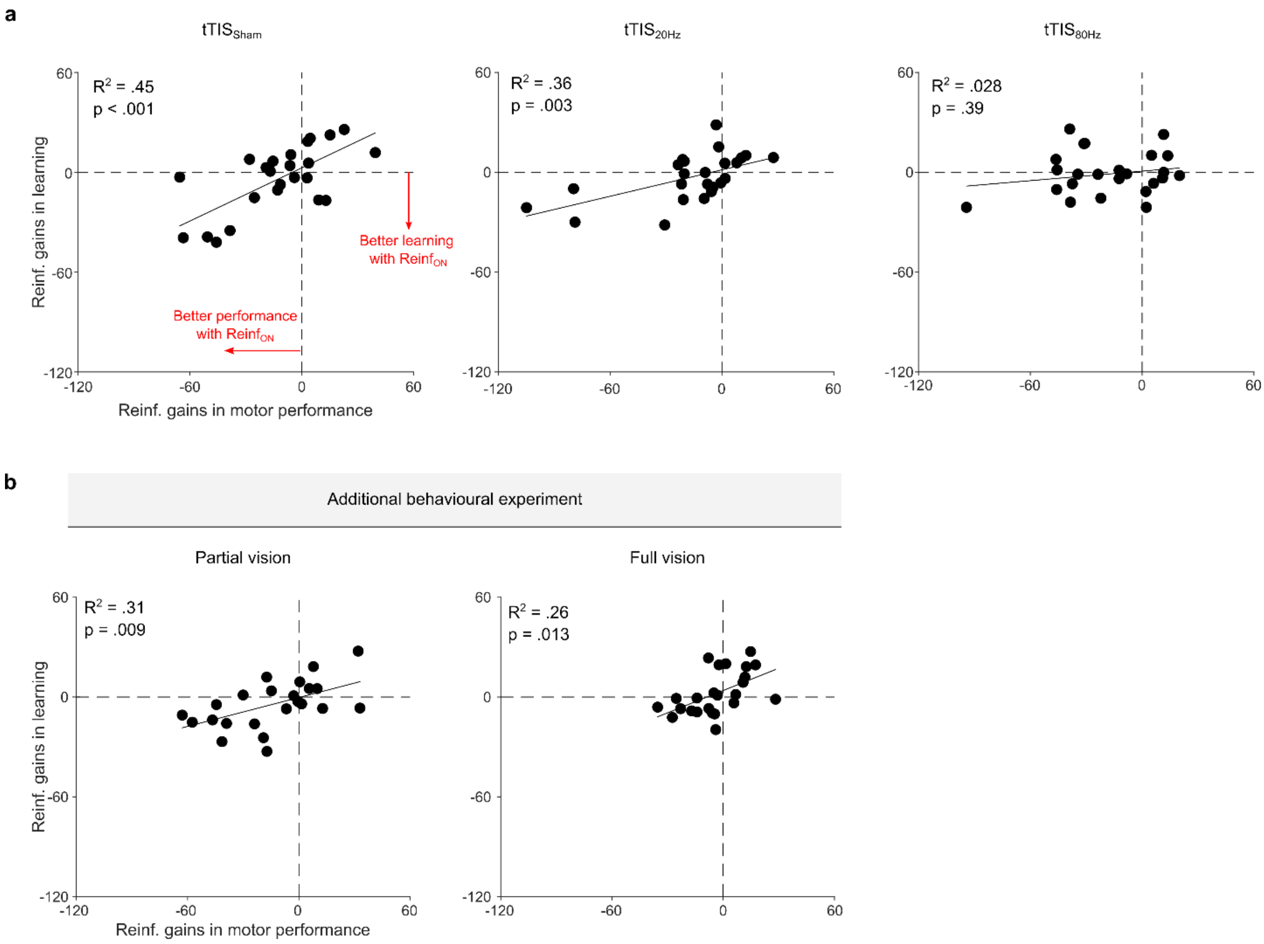

Figure S2. Correlation between reinforcement gains in Training (T6) and at Post-training (Post1). a) Robust linear regressions in the three tTIS conditions of the main study are presented with the statistics of the corresponding robust linear regressions (n = 24). b) Replication of these correlations in the control behavioural dataset (n = 24 independent subjects) for partial vision of the cursor (left part, same condition as in the tTIS study) and full vision of the cursor (right part).

6. Control analyses of behavioural data

Pre-training performance

In order to verify that our main behavioural results were not influenced by potential differences in initial performance between conditions despite randomisation, we analysed the Error at Pre-training between conditions. We did not find any $tTIS_{TYPE}$ ($F_{(2,519.15)}$=1.64; p=0.20, η_p^2=0.006, 95% CI [0.00, 0.02]) or $tTIS_{TYPE}$ x $Reinf_{TYPE}$ effect ($F_{(2,519.99)}$=1.08; p=0.34, η_p^2=0.004, 95% CI [0.00, 0.02]), suggesting that the main behavioural results could not be accounted for by differences in initial performance between conditions. However, the LMM did reveal a $Reinf_{TYPE}$ effect ($F_{(1,519.15)}$=12.47; p<0.001, η_p^2=0.02, 95% CI [0.00, 0.06]), that was due to the fact that Pre-training performance was generally better in $Reinf_{OFF}$ blocks. This effect, which was opposite to our learning results (generally better learning with $Reinf_{ON}$), may be related to an expectancy effect stemming from the repetitive structure of the reinforcement conditions (see Methods). However, the absence of interaction with $tTIS_{TYPE}$ strongly suggests that this effect did not drive any of the main findings. Put together, these data provide confidence that the differential effects of striatal tTIS on motor learning depending on the presence of reinforcement were not the result of different initial performance between conditions.

Success rate

Overall, the amount of positive reinforcement (i.e., when the target was green) averaged 52.78 +/- 0.42% and did not differ depending on the $tTIS_{TYPES}$ ($F_{(2,1702)}$=0.17; p=0.84, η_p^2=2x10^{-4}) suggesting that the closed-loop reinforcement schedule was successful at providing comparable reinforcement feedback despite differences in performance between conditions. Hence, different success rates during training cannot explain the effect of the different striatal tTIS conditions on motor learning.

Frequency of flashing

Analysis of the frequency of flashing in the different conditions did not reveal any effect of $tTIS_{TYPE}$ ($F_{(2,3283)}$=0.85; p=0.43, η_p^2=5x10^{-4}) nor any $Reinf_{TYPE}$ x $tTIS_{TYPE}$ interaction ($F_{(2,3283)}$=0.19; p=0.82, η_p^2=1x10^{-4}), suggesting that the behavioural effects of tTIS could not be explained by a visual confound. However, this analysis did reveal a $Reinf_{TYPE}$ effect ($F_{(1,3283)}$=33.62; p<0.001, η_p^2=0.01, 95% CI [0.00, 0.02]) which was due to the fact that the

average frequency in the $\mathrm{Reinf_{OFF}}$ condition (4.28 ± 0.097 Hz) was slightly but significantly higher than with $\mathrm{Reinf_{ON}}$ (4.08 ± 0.098 Hz). Notably, in absolute terms, this difference represented only a difference of 1.4 change of color over the whole 7 s trial, which we think is unlikely to explain the improvement of performance in the $\mathrm{Reinf_{ON}}$ condition.

Order of the reinforcement conditions

Previous exposure to reinforcement feedback may improve subsequent learning through reinforcement[4]. Thanks to our randomisation procedure, the previous exposure to the $\mathrm{Reinf_{ON}}$ condition was equally counterbalanced in all stimulation conditions, and should therefore not influence our main results. Still, we performed an analysis to specifically investigate the effect of the previous exposure to $\mathrm{Reinf_{ON}}$. To do so, we split the participants depending on whether they experienced $\mathrm{Reinf_{ON}}$ or $\mathrm{Reinf_{OFF}}$ first (12 subjects per group) and performed a new LMM on the Post-training data with the addition of a categorical factor $\mathrm{Group_{TYPE}}$. In particular, if the previous exposure to the $\mathrm{Reinf_{ON}}$ condition influenced following learning with reinforcement, we would expect to see a $\mathrm{Group_{TYPE}}$ x $\mathrm{Reinf_{TYPE}}$ interaction. The analysis did not indicate any $\mathrm{Group_{TYPE}}$ effect on learning ($F_{(1,21.96)}$=0.35; p=0.56, η_p^2=0.02, 95% CI [0.00, 0.23]), neither did it reveal a $\mathrm{Group_{TYPE}}$ x $\mathrm{Reinf_{TYPE}}$ ($F_{(1,4)}$=0.72; p=0.44, η_p^2=0.15, 95% CI [0.00, 0.67]), or a triple interaction with $\mathrm{tTIS_{TYPE}}$ ($F_{(2,1105.06)}$=1.75; p=0.17, η_p^2=0.003, 95% CI [0.00, 0.01]). Overall, this analysis suggests that the order of the exposure to the reinforcement condition did not influence the present findings.

7. Blinding integrity and tTIS-evoked sensations

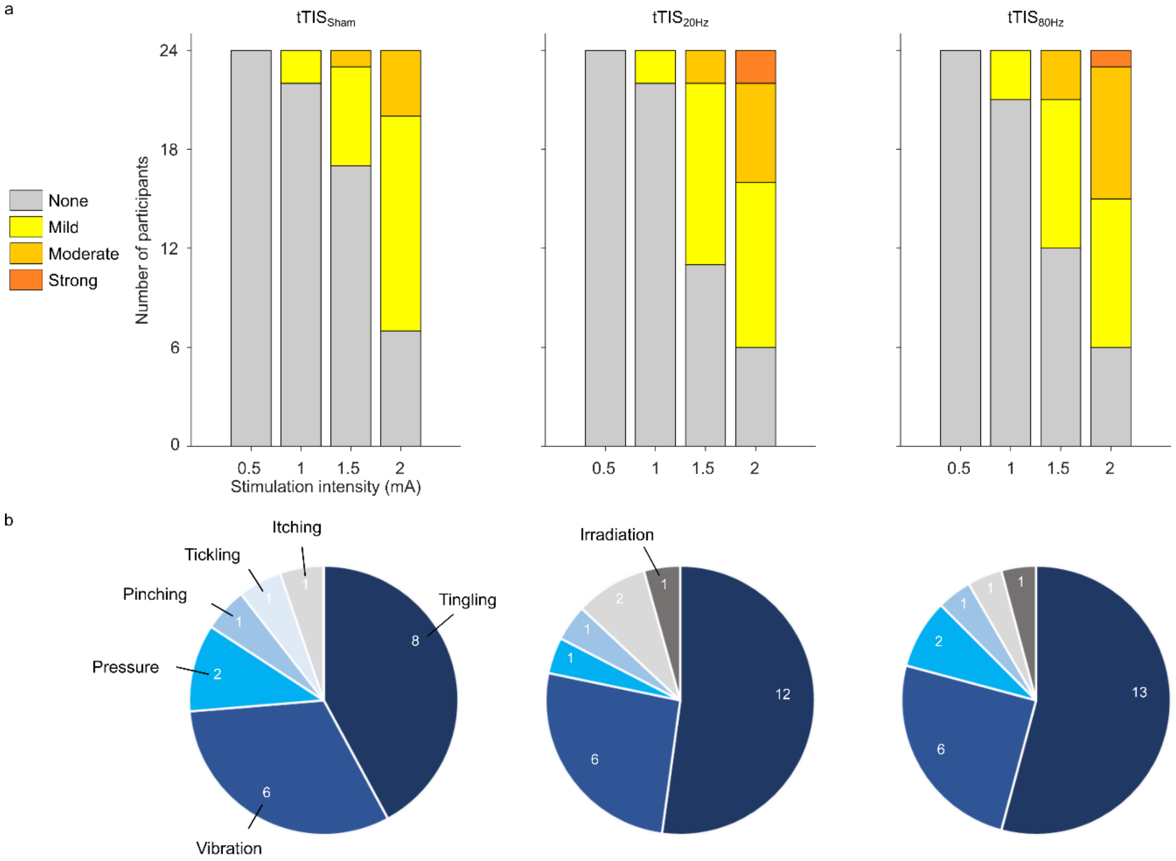

Figure S3. tTIS-related sensations. a) Magnitude of tTIS-related sensations. Magnitude of sensations reported before the experiment for current amplitudes ranging from 0.5 to 2 mA for each $tTIS_{TYPE}$. The current amplitude used in the present experiment was 2 mA. **b) Types of tTIS-related sensations.** Type of sensations as described by the participants, at 2 mA. Note that subjects were allowed to describe their sensations with up to two different words.

8. Brain activity during reinforcement motor learning

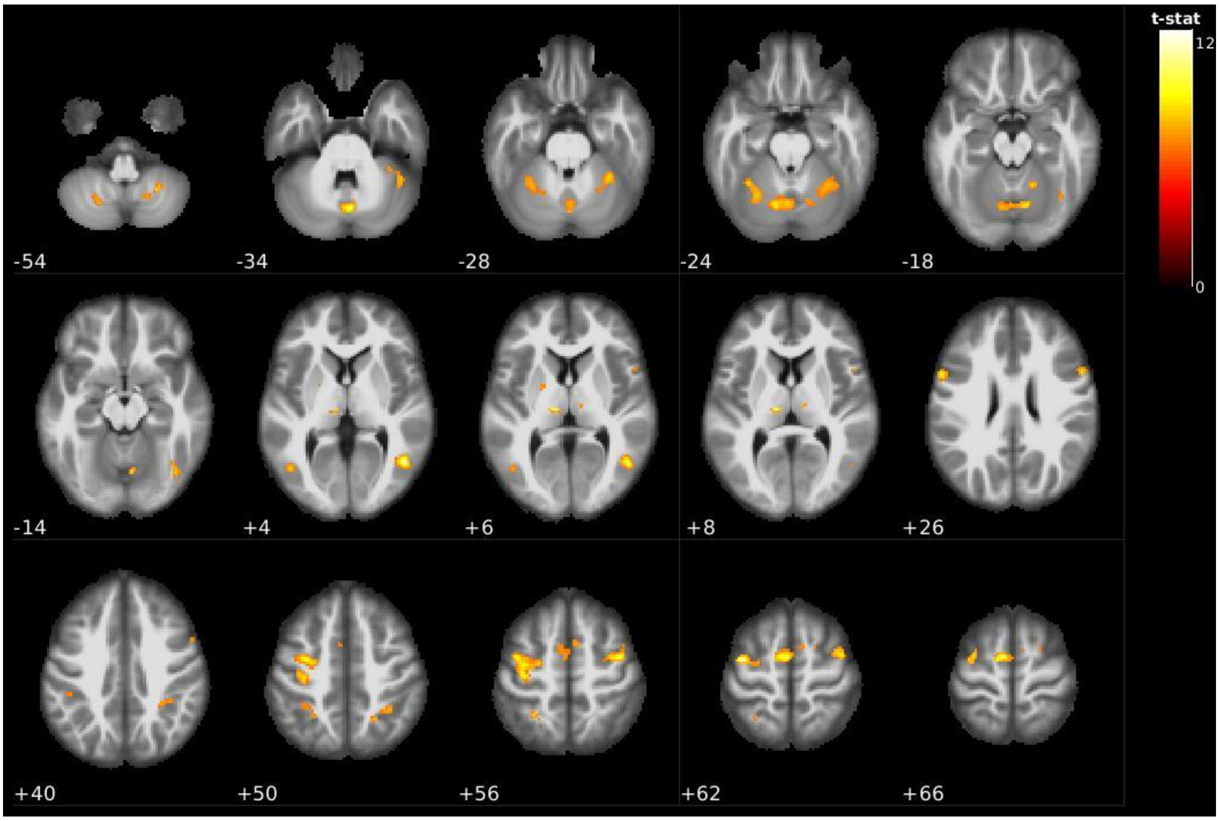

Figure S4. Whole-brain activity during reinforcement motor learning. Activation maps for the contrast task>rest in the $tTIS_{Sham}$, $Reinf_{ON}$ condition showing activation of key areas of the reinforcement motor learning network including the putamen, thalamus, cerebellum and sensorimotor network, especially on the left side. Significant clusters in the t-contrast are shown for corrected voxel-wise family wise error (FWE), p=0.05, and corrected cluster-based false discovery rate (FDR), p=0.05.

Cluster-level				Peak-level					x	y	z	Region
$p_{FWE-corr}$	$q_{FDR-corr}$	k_E	p_{uncorr}	$p_{FWE-corr}$	$q_{FDR-corr}$	T	(Z_E)	p_{uncorr}				
<0.001	<0.001	135	<0.001	<0.001	0.005	12.63	6.84	<0.001	46	-62	4	Temporal_Mid_R
<0.001	<0.001	523	<0.001	<0.001	0.005	12.32	6.77	<0.001	-40	-8	62	Precentral_L
				<0.001	0.021	10.62	6.33	<0.001	-34	-6	52	Postcentral_L
				<0.001	0.021	10.43	6.28	<0.001	-36	-20	54	Precentral_L
<0.001	<0.001	335	<0.001	<0.001	0.018	11.08	6.46	<0.001	-8	-6	64	Supp_Motor_Area_L
				0.003	0.145	8.21	5.56	<0.001	6	6	58	Supp_Motor_Area_R
				0.003	0.145	8.20	5.55	<0.001	-4	-2	54	Supp_Motor_Area_L
<0.001	<0.001	44	<0.001	<0.001	0.021	10.65	6.34	<0.001	-10	-20	6	Thal_IL_L
<0.001	<0.001	162	<0.001	<0.001	0.021	10.36	6.26	<0.001	42	-6	56	Frontal_Mid_2_R
				<0.001	0.042	9.48	5.99	<0.001	34	-4	58	Frontal_Sup_2_R
<0.001	<0.001	175	<0.001	<0.001	0.021	10.27	6.23	<0.001	-58	10	28	Precentral_L
				<0.001	0.037	9.60	6.03	<0.001	-56	8	20	Frontal_Inf_Oper_L
				0.019	0.490	7.32	5.21	<0.001	-48	2	16	Rolandic_Oper_L
<0.001	<0.001	601	<0.001	<0.001	0.024	10.06	6.17	<0.001	2	-74	-34	Vermis_7
				<0.001	0.025	9.99	6.15	<0.001	-12	-70	-22	Cerebellum_6_L
				<0.001	0.027	9.88	6.12	<0.001	12	-70	-20	Cerebellum_6_R
<0.001	<0.001	82	<0.001	<0.001	0.070	9.14	5.88	<0.001	56	10	26	Frontal_Inf_Oper_R
				0.006	0.234	7.86	5.42	<0.001	56	10	38	Precentral_R
<0.001	<0.001	141	<0.001	0.001	0.092	8.89	5.80	<0.001	-34	-52	-24	Cerebellum_6_L
				0.002	0.117	8.47	5.65	<0.001	-28	-62	-24	Cerebellum_6_L
<0.001	<0.001	76	<0.001	0.001	0.092	8.87	5.79	<0.001	-28	-52	56	Parietal_Sup_L
				0.011	0.341	7.57	5.31	<0.001	-30	-44	48	Parietal_Inf_L
<0.001	<0.001	200	<0.001	0.001	0.092	8.77	5.76	<0.001	32	-48	-28	Cerebellum_6_R
				0.013	0.382	7.49	5.28	<0.001	34	-40	-34	Cerebellum_6_R
<0.001	<0.001	36	<0.001	0.001	0.092	8.73	5.74	<0.001	16	-54	-18	Cerebellum_4_5_R
<0.001	<0.001	28	<0.001	0.001	0.101	8.63	5.71	<0.001	26	-58	-54	Cerebellum_8_R
<0.001	<0.001	62	<0.001	0.001	0.113	8.51	5.67	<0.001	38	-62	-16	Fusiform_R
				0.002	0.117	8.45	5.64	<0.001	42	-72	-12	Occipital_Inf_R
<0.001	<0.001	21	<0.001	0.002	0.117	8.41	5.63	<0.001	-46	-68	4	Occipital_Mid_L
<0.001	<0.001	141	<0.001	0.002	0.130	8.33	5.60	<0.001	22	-56	50	Location not in atlas
				0.002	0.130	8.30	5.59	<0.001	30	-48	48	Parietal_Sup_R
				0.007	0.266	7.76	5.39	<0.001	36	-40	42	SupraMarginal_R
<0.001	<0.001	29	<0.001	0.004	0.170	8.09	5.51	<0.001	44	-50	-34	Cerebellum_Crus1_R
<0.001	<0.001	59	<0.001	0.004	0.178	8.04	5.49	<0.001	-22	-66	-52	Cerebellum_8_L
<0.001	0.006	12	0.003	0.004	0.190	7.99	5.47	<0.001	10	-16	8	Thal_MDl_R
0.001	0.043	6	0.028	0.009	0.319	7.63	5.33	<0.001	-22	-2	6	Putamen_L
<0.001	<0.001	34	<0.001	0.009	0.319	7.63	5.33	<0.001	18	-64	-54	Cerebellum_8_R
0.001	0.300	7	0.019	0.023	0.545	7.23	5.17	<0.001	20	2	62	Frontal_Sup_2_R
0.001	0.030	7	0.019	0.024	0.560	7.21	5.16	<0.001	52	12	8	Frontal_Inf_Oper_R
0.001	0.030	7	0.019	0.025	0.568	7.19	5.16	<0.001	-44	-36	40	Parietal_Inf_L

Table S1: Significant clusters and the respective local maxima in the $tTIS_{Sham}$, $Reinf_{ON}$ condition. Related to Figure S4. Regions were identified with the Automated Anatomical Labelling atlas 3 (AAL3[5]). Significant clusters in the t-contrast were selected for

corrected voxel-wise family wise error (FWE), p=0.05, and corrected cluster-based false discovery rate (FDR), p=0.05.

9. Correlation between effect of $tTIS_{80Hz}$ on reinforcement motor learning and modulation of whole-brain activity

Cluster-level				Peak-level					x	y	z	Region
$p_{FWE\text{-}corr}$	$q_{FDR\text{-}corr}$	k_E	P_{uncorr}	$p_{FWE\text{-}corr}$	$q_{FDR\text{-}corr}$	T	(Z_E)	P_{uncorr}				
**0.003**	**0.005**	**157**	**<0.001**	**0.027**	**0.065**	**7.29**	**5.14**	**<0.001**	**10**	**18**	**0**	Caudate_R
				0.639	0.678	5.38	4.25	<0.001	0	0	10	Location not in atlas
				0.921	0.757	4.89	3.98	<0.001	6	6	2	Location not in atlas
**0.007**	**0.005**	**138**	**<0.001**	**0.693**	**0.678**	**5.30**	**4.21**	**<0.001**	**-16**	**14**	**6**	Location not in atlas
				0.923	0.757	4.88	3.98	<0.001	-22	14	-2	Putamen_L
				1.000	0.810	4.26	3.60	<0.001	-18	8	-6	Putamen_L

Table S2. Significant clusters for the correlation between the behavioural and neural effects of $tTIS_{80Hz}$ (vs. $tTIS_{20Hz}$). Related to Figure 3b. Two significant clusters were found with several local maxima. Notably, the left cluster also encompassed a portion of the left caudate (related to Figure 3). Regions were identified with the Automated Anatomical Labelling atlas 3 (AAL3[5]). Significant clusters in the t-contrast were selected for uncorrected p=0.001 at the voxel level, and corrected cluster-based false discovery rate (FDR), p=0.05.

10. Control analysis on striatum to frontal cortex effective connectivity

The connectivity analysis showed that $tTIS_{80Hz}$, but not $tTIS_{20Hz}$, increased striatum to frontal effective connectivity and that this effect depended on the type of network considered (reward vs. motor) and on the presence of reinforcement (Figure 4). In this analysis we considered effective connectivity between the motor striatum and M1 and SMA for the motor network and the limbic striatum with ACC and vmPFC for the reward network, based on a large body of literature[6–9] (see Methods for a detailed justification of the ROIs). To verify whether our results depended on the specific frontal ROIs included in the analysis, we performed a new analysis. More specifically, we decomposed connectivity in each network for each frontal cortical area (M1 and SMA in the motor network and ACC and vmPFC in the reward network) and ran two separate LMMs on each network with $tTIS_{TYPE}$, $Reinf_{TYPE}$ as well as ROI_{TYPE} (M1

or SMA for the LMM run on the motor network and ACC or vmPFC for the reward network) as fixed effects. Consistent with our initial findings, we found effects of $tTIS_{TYPE}$ on both LMMs (motor network: $F_{(2,1089.7)}$=3.12; p=0.044, η_p^2=0.006, 95% CI [0.00, 0.02] and reward network: $F_{(2,1112)}$=6.78; p=0.001, η_p^2=0.01, 95% CI [0.00, 0.03]). Moreover, there was a significant $tTIS_{TYPE}$ x $Reinf_{TYPE}$ interaction in the motor network ($F_{(2,1112)}$=3.36; p=0.035, η_p^2=0.006, 95% CI [0.00, 0.02]), which was not significant in the reward network ($F_{(2,1113.8)}$=2.37; p=0.094, η_p^2=0.004, 95% CI [0.00, 0.01]). Most importantly, these effects were not modulated by ROI_{TYPE} in any network ($tTIS_{TYPE}$ x $Reinf_{TYPE}$ x ROI_{TYPE} in motor network: $F_{(2,1112)}$=0.83; p=0.44, η_p^2=0.001, 95% CI [0.00, 0.01], in reward network: $F_{(2,1112)}$=0.61; p=0.54, η_p^2=0.001, 95% CI [0.00, 0.01]). This analysis suggests that the main connectivity findings were not influenced by the specific frontal ROIs considered in the analysis.

11. Relationship between the neural and behavioural effects of $tTIS_{80Hz}$ and impulsivity

Characterising individual factors that influence responsiveness to brain stimulation is an important line of research both for fundamental neuroscience but also to determine profiles of responders for future clinical translation. Based on previous literature linking striatal gamma oscillatory mechanisms and impulsivity[10], we explored the possibility that impulsivity influences responsiveness to striatal $tTIS_{80Hz}$ (**Figure S5**).

First, we exploited the BOLD data and asked if inter-individual variability in the neural effects of $tTIS_{80Hz}$ during reinforcement motor learning (i.e., in the $Reinf_{ON}$ condition) was related to impulsivity at the whole-brain level. Impulsivity was evaluated by a well-established independent delay-discounting questionnaire performed at the beginning of the experiment[11,12]. Strikingly, this analysis revealed that impulsivity was associated to the effect of $tTIS_{80Hz}$ (with respect to $tTIS_{20Hz}$) specifically in the left caudate nucleus (Figure S5a, Table S3). No other clusters were found. As such, the most impulsive participants exhibited an increase of left caudate activity with $tTIS_{80Hz}$ (compared to $tTIS_{20Hz}$) while the least impulsive ones rather

presented a decrease of BOLD signal, consistent with the idea that impulsivity modulates the neuronal responsiveness to tTIS (R^2=0.47; p<0.001; Figure S5b). No significant clusters of correlation were found for the $tTIS_{80Hz}$ – $tTIS_{Sham}$ contrast, neither for the control $tTIS_{20Hz}$ - $tTIS_{Sham}$ contrast. Hence, this analysis suggests that the effect of $tTIS_{80Hz}$ on caudate activity depends on participants' impulsivity.

As a second step, we aimed at evaluating the association between impulsivity and the increased striatum to motor cortex connectivity observed with $tTIS_{80Hz}$, in the presence of reinforcement. Notably, such pattern of increased connectivity in fronto-striatal circuits has been described as a pathophysiological mechanism in multiple neuro-psychiatric disorders involving impulsivity[13–16]. Hence, we first asked if striatum to motor cortex connectivity was related to impulsivity during reinforcement motor learning in the absence of stimulation (i.e., in the $tTIS_{Sham}$ condition). Indeed, we found a significant positive relationship between impulsivity and striatum to motor cortex connectivity (robust linear regression: R^2=0.10; p=0.004), in line with previous results[13–16]. Then, we evaluated whether the increase of connectivity observed with $tTIS_{80Hz}$ in the $Reinf_{ON}$ condition (Figure 4a) could be related to impulsivity. Indeed, we found that the effect of $tTIS_{80Hz}$ on connectivity was negatively correlated to impulsivity both when contrasting $tTIS_{80Hz}$ with $tTIS_{Sham}$ (R^2=0.19; p=0.043, Figure S5c, left) and with $tTIS_{20Hz}$ (R^2=0.28; p=0.021, Figure S5c, middle): participants with the largest increase in connectivity with $tTIS_{80Hz}$ in the $Reinf_{ON}$ condition were also the least impulsive ones. Such correlation was absent when contrasting $tTIS_{20Hz}$ and $tTIS_{Sham}$ (R^2=0.0031; p=0.31, Figure S5C, right), but also when considering the same contrasts in the reward instead of the motor network (p=0.93 and p=0.86 for the $tTIS_{80Hz}$-$tTIS_{Sham}$ and $tTIS_{80Hz}$-$tTIS_{20Hz}$ contrasts, respectively). Hence, striatum to motor cortex effective connectivity during the task was positively correlated to impulsivity, but the change in connectivity induced by $tTIS_{80Hz}$ was rather negatively associated with impulsivity. This may be due to a ceiling effect in the most impulsive participants: exhibiting initially high levels of connectivity may leave less room for further modulation by $tTIS_{80Hz}$. These

results suggest that inter-individual variability in impulsivity might influence neural responses to striatal $tTIS_{80Hz}$.

As a last step, we verified if impulsivity was also predictive of the behavioural effects of $tTIS_{80Hz}$ on reinforcement motor learning. We did not find any significant correlation between impulsivity and the effect of $tTIS_{80Hz}$ on motor learning ($tTIS_{80Hz}$ – $tTIS_{Sham}$: R^2=0.098; p=0.17; $tTIS_{80Hz}$ – $tTIS_{20Hz}$: R^2=0.11; p=0.21). Hence, impulsivity was associated to the neural, but not the behavioural effects of $tTIS_{80Hz}$.

Overall, we found that impulsivity was associated to $tTIS_{80Hz}$-related BOLD changes specifically in the left caudate and to changes of effective connectivity between the motor striatum and motor cortex during reinforcement motor learning. Hence, a possibility is that the differences in endogenous striatal gamma-related activity that have been associated to impulsive behaviour in animal models[10,17,18], influence the neural effects of $tTIS_{80Hz}$. If this is the case, impulsivity could constitute a behavioural factor allowing to determine responsiveness to striatal $tTIS_{80Hz}$. Conversely, an interesting avenue for future research could aim at determining whether impulsivity can be modulated by striatal $tTIS_{80Hz}$.

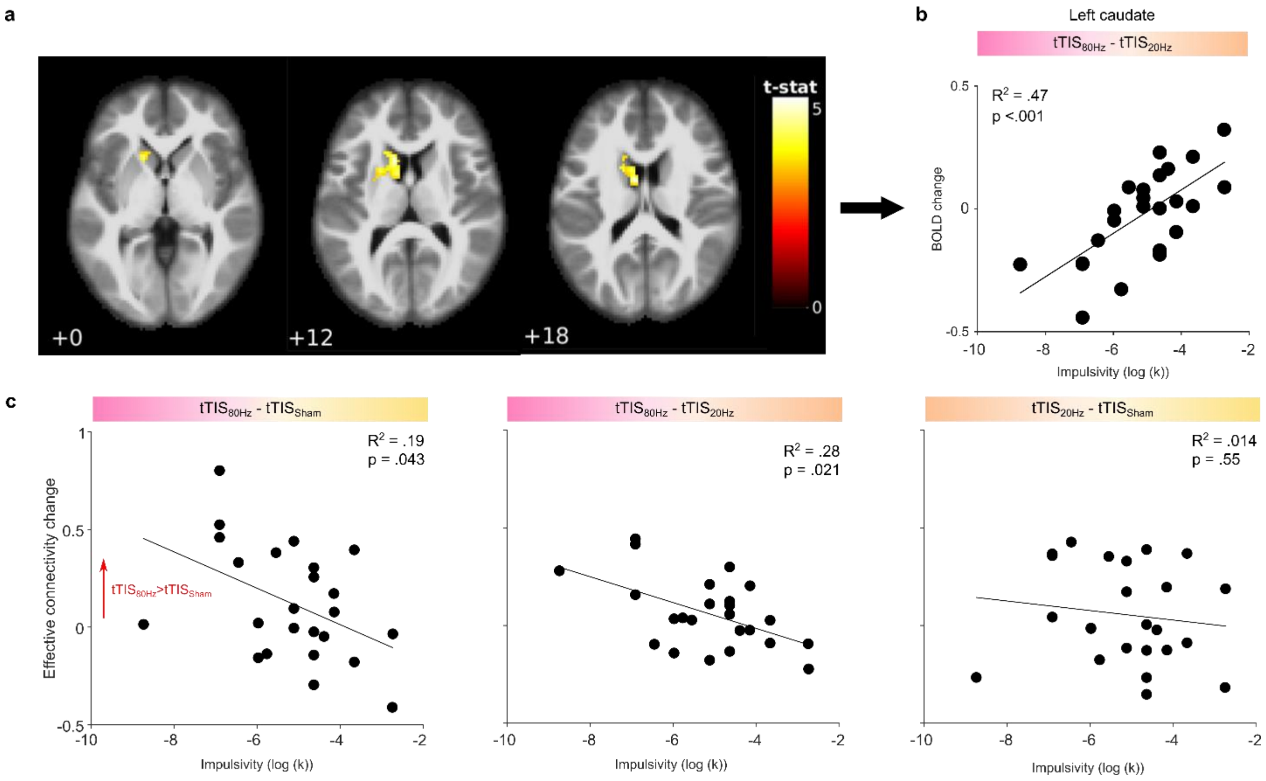

Figure S5. Relationship between impulsivity and the neural effects of $tTIS_{80Hz}$. a) Whole-brain correlation between the neural effects of $tTIS_{80Hz}$ (with respect to $tTIS_{20Hz}$) and impulsivity. Correlation between tTIS-related modulation of striatal activity ($tTIS_{80Hz}$ – $tTIS_{20Hz}$) during reinforcement motor learning ($Reinf_{ON}$) and individual impulsivity levels. A single significant cluster of correlation was found in left caudate (t-contrast, uncorrected p=0.001 at the voxel level, and corrected cluster-based FDR: p=0.05). **b) Correlation between left caudate activity and impulsivity.** A positive correlation was found showing that participants with higher levels of impulsivity exhibited stronger activation of the left caudate in the $tTIS_{80Hz}$ (with respect to $tTIS_{20Hz}$, robust linear regression). **c) Correlations between impulsivity and tTIS-related modulation of effective connectivity.** Impulsivity was associated to the neural effects of $tTIS_{80Hz}$ both when contrasting to $tTIS_{Sham}$ (left) and $tTIS_{20Hz}$ (middle), but was not correlated to the effect of $tTIS_{20Hz}$ (right, robust linear regressions).

Cluster-level				Peak-level					x	y	z	Region
$p_{FWE\text{-}corr}$	$q_{FDR\text{-}corr}$	k_E	P_{uncorr}	$p_{FWE\text{-}corr}$	$q_{FDR\text{-}corr}$	T	(Z_E)	P_{uncorr}				
**<0.001**	**<0.001**	**254**	**<0.001**	**0.707**	**0.524**	**5.29**	**4.20**	**<0.001**	**-8**	**0**	**18**	Location not in atlas
				0.719	0.524	5.27	4.19	<0.001	-14	16	16	Caudate_L
				0.971	0.620	4.72	3.88	<0.001	-16	16	0	Location not in atlas

Table S3. Significant clusters for the correlation between impulsivity and effects of $tTIS_{80Hz}$ on BOLD activity (vs. $tTIS_{20Hz}$). Related to Figure S5a. One significant cluster encompassing the left caudate nucleus was found (t-contrast, uncorrected p=0.001 at the voxel level, and corrected cluster-based false discovery rate (FDR), p=0.05). Regions were identified with AAL3[5].

12. <u>Exclusion criteria</u>

- Unable to consent
- Severe neuropsychiatric (e.g., major depression, severe dementia) or unstable systemic diseases (e.g., severe progressive and unstable cancer, life threatening infectious diseases)
- Severe sensory or cognitive impairment or musculoskeletal dysfunctions prohibiting to understand instructions or to perform the experimental tasks
- Color blindness
- Inability to follow or non-compliance with the procedures of the study
- Contraindications for NIBS or MRI:
  - Electronic or ferromagnetic medical implants/device, non-MRI compatible metal implant
  - History of seizures
  - Medication that significantly interacts with NIBS being benzodiazepines, tricyclic antidepressant and antipsychotics
- Regular use of narcotic drugs
- Left-handedness
- Pregnancy
- Request of not being informed in case of incidental findings

- Concomitant participation in another trial involving probing of neuronal plasticity.

13. ContES Checklist

**Technological factors**	
Manufacturer of Stimulator	DS5 Isolated Bipolar Constant Current Stimulator (Digitimer)
MR Conditional Electrode Details	Round, 3 cm^2 conductive rubber electrodes
Electrode Positioning	F3 → F4<br>TP7 → TP8<br>A bandage is warped around the head to apply pressure and keep the electrodes in place<br>Electrodes are oriented in order to have vertical cables entering parallel to the MRI coil<br>Head was fixed with pillows to avoid movements
MR Conditional Skin-Electrode Interface	10-20 gel<br>One or two drops of saline were added when impedances were too high
Amount of Contact Medium (Paste/Gel/Electrolyte)	Around 1mm of paste was manually placed on the electrodes
Electrode Placement Visualization	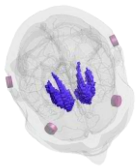
RF Filter	NeuroConn DC-STIMULATOR MR RF filter module with MRI-compatible cables and electrodes
Wire Routing Pattern	10 m ethernet cables between inner and outer box pass through a conduit along the wall of the MRI room until reaching the back of the MRI. Cables are then fixed with straps on the ground and on the wall of the MRI machine in order to avoid loops until reaching the interior of the coil.<br>Cables between the head and the inner boxes were also fixed with straps and they were oriented in order to exit the magnetic field direction as soon as possible as indicated by the red arrows of the image below.<br>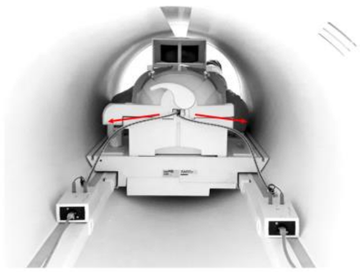

tES-fMRI Machine Synchronization/Communication	Stimulation was triggered by the stimulus delivery PC via parallel port to BNC cable. The parallel port of the stimulus delivery PC was connected to the DAQ controlling the stimulators.<br>Stimulus delivery PC, in turn, was also receiving the scanner trigger from the scanner via USB port.
**Safety and noise tests**	
MR Conditionality Specifics for tES Setting	Please refer to Section "Methods-Imaging acquisition"
tES-fMRI Setting Test - Safety Testing	Impedances were checked before and after the stimulation.<br>No temperature tests were performed during the experiment.<br>Intensity titration was performed prior to entering the MRI, testing increasing currents (0.5, 1, 1.5 and 2 mA) and asking the subject to report any type of sensation.<br>A sensation questionnaire was also performed at the end of the experiment.
tES-fMRI Setting Test - Subjective Intolerance Reporting	No intolerances were reported by any subject
tES-fMRI Setting Test - Noise/Artifact	Signal to Noise Ratio (SNR) analysis was performed on the fMRI images, please refer to Section "Methods-Signal to Noise Ratio"
Impedance Testing	Impedances were checked right after electrodes positioning outside the scanner, before and after the stimulation inside.<br>One or two drops of saline solution were added if impedances were higher than 20kΩ
**Methodological factors**	
Concurrent tES-fMRI Timing	For timings, please refer to the "Methods-Stimulation protocols" section<br>To mitigate the impact of potential carry-over effects on our experimental results we used the following strategy:<br>1) We stimulated for short periods in each condition (5 minutes interspersed with resting periods without stimulation; see "Methods-Stimulation protocols");<br>2) We imposed breaks (~7-8 minutes) between each stimulation protocol;<br>3) We randomised the order of the Stimulation conditions
Imaging Session Timing	All sequences were performed with TI stimulation electrodes placed on the subjects' head.
tES Experience Report	Please refer to "Results" section and to Figure S3.

Table S4. ContES checklist as recommended in Ekhtiari et al., 2022[19] for concurrent tES-fMRI studies.

14. Imaging quality control

A threshold of 0.5 was chosen to discard subjects showing more than 40% of voxels with framewise displacement FD higher than this threshold. In the current study cohort, no subject exceeded the limit value, thus the whole dataset could be used. Furthermore, successful cleaning of the data was ensured by visual checking the preprocessing results. In particular, good registration between anatomical and functional images and normalization to standard space were checked. Signal to noise ratio analysis showed significantly higher tSNR values underneath the stimulating electrodes ($F_{(1,1122)}$=249.25, $p<0.001$; **Figure S6**). Moreover, there was no evidence for a modulation of this effect by the $tTIS_{TYPE}$ ($Sphere_{LOCATION}$ x $tTIS_{TYPE}$: $F_{(2,1118)}$=0.0169, p=0.98). This result suggests that the stimulation did not introduce additional noise to the MR images. In summary, all controls confirmed the good quality of the imaging data.

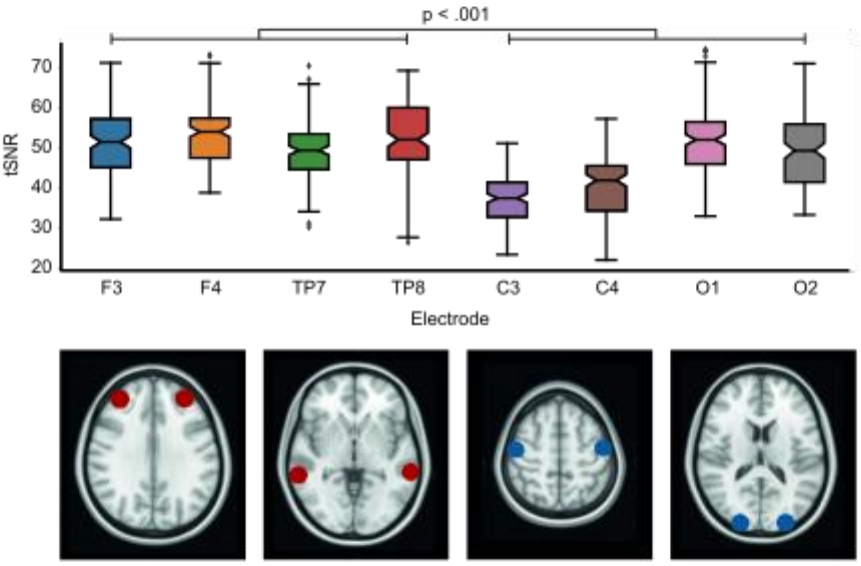

Figure S6. Total signal to noise ratio (tSNR). Total signal to noise ratio investigation. On the top panel, the average tSNR is shown within spheres of 10mm radius underneath the 4 stimulation electrodes (F3, F4, TP7 and TP8) and underneath other 4 locations more distal from the electrodes (C3, C4, O1 and O2). A significant higher tSNR was found underneath the electrodes with respect to the distal locations (one-sided ANOVA with Satterthwaite's approximations: $F_{(1,1122)}$=249.25, $p<0.001$, n = 24 subjects, 6 scans each). This indicates that there was no reduction of the tSNR due to the presence of electrical current. In all box plots, the crossbar represents the median value, colored areas represent the interquartile range, and the whiskers represent the 1.5 interquartile range. Diamonds show individual points outside the 1.5 interquartile range. On the bottom panel, the location of the spheres from where the average tSNRs were extracted: F3 and F4 in red in the first image from the left, TP7 and TP8 in red on the second image from the left, C3 and C4 in blue on the third image from the left, O1 and O2 in blue on the fourth image from the left.

References

1. Izawa, J. & Shadmehr, R. Learning from sensory and reward prediction errors during motor adaptation. *PLoS Comput Biol* **7**, 1–12 (2011).
2. Cashaback, J. G. A., McGregor, H. R., Mohatarem, A. & Gribble, P. L. Dissociating error-based and reinforcement-based loss functions during sensorimotor learning. *PLoS Comput Biol* **13**, 1–28 (2017).
3. Lam, S. Y. & Zénon, A. Information rate in humans during visuomotor tracking. *Entropy* **23**, 1–13 (2021).
4. Uehara, S., Mawase, F., Therrien, A. S., Cherry-Allen, K. M. & Celnik, P. Interactions between motor exploration and reinforcement learning. *J Neurophysiol* **122**, 797–808 (2019).
5. Rolls, E. T., Huang, C. C., Lin, C. P., Feng, J. & Joliot, M. Automated anatomical labelling atlas 3. *Neuroimage* **206**, 116189 (2020).
6. Hardwick, R. M., Rottschy, C., Miall, R. C. & Eickhoff, S. B. A quantitative meta-analysis and review of motor learning in the human brain. *Neuroimage* **67**, 283–297 (2013).
7. Bartra, O., McGuire, J. T. & Kable, J. W. The valuation system: A coordinate-based meta-analysis of BOLD fMRI experiments examining neural correlates of subjective value. *Neuroimage* **76**, 412–427 (2013).
8. Draganski, B. *et al.* Evidence for segregated and integrative connectivity patterns in the human basal ganglia. *Journal of Neuroscience* **28**, 7143–7152 (2008).
9. Morris, L. S. *et al.* Fronto-striatal organization: Defining functional and microstructural substrates of behavioural flexibility. *Cortex* **74**, 118–133 (2016).
10. Donnelly, N. A. *et al.* Oscillatory activity in the medial prefrontal cortex and nucleus accumbens correlates with impulsivity and reward outcome. *PLoS One* **9**, 14–17 (2014).
11. Kirby, K. N., Petry, N. M. & Bickel, W. K. Heroin addicts have higher discount rates for delayed rewards than non-drug-using controls. *J Exp Psychol Gen* **128**, 78–87 (1999).
12. Mitchell, J. M., Fields, H. L., D'Esposito, M. & Boettiger, C. A. Impulsive responding in alcoholics. *Alcohol Clin Exp Res* **29**, 2158–2169 (2005).
13. Hampton, W. H., Alm, K. H., Venkatraman, V., Nugiel, T. & Olson, I. R. Dissociable frontostriatal white matter connectivity underlies reward and motor impulsivity. *Neuroimage* **150**, 336–343 (2017).
14. Mosley, P. E. *et al.* The structural connectivity of discrete networks underlies impulsivity and gambling in Parkinson's disease. *Brain* **142**, 3917–3935 (2019).
15. Ma, I. *et al.* Ventral striatal hyperconnectivity during rewarded interference control in adolescents with ADHD. *Cortex* **82**, 225–236 (2016).
16. Wang, Q. *et al.* Dissociated neural substrates underlying impulsive choice and impulsive action. *Neuroimage* **134**, 540–549 (2016).
17. Pisansky, M. T. *et al.* Nucleus Accumbens Fast-Spiking Interneurons Constrain Impulsive Action. *Biol Psychiatry* **86**, 836–847 (2019).
18. Schall, T. A., Wright, W. J. & Dong, Y. Nucleus accumbens fast-spiking interneurons in motivational and addictive behaviors. *Mol Psychiatry* **26**, 234–246 (2021).
19. Ekhtiari, H. *et al.* A checklist for assessing the methodological quality of concurrent tES-fMRI studies (ContES checklist): a consensus study and statement. *Nat Protoc* **17**, 596–617 (2022).
