# Supplementary material for: Non-invasive stimulation of the human striatum disrupts reinforcement learning of motor skills
Source: Nat Hum Behav. 2024 May 29;8(8):1581–98. doi: 10.1038/s41562-024-01901-z (PMC11343719; doi:10.1038/s41562-024-01901-z)
Supplement: Supplementary file 2 — Reporting Summary [file 41562_2024_1901_MOESM2_ESM.pdf]

nature portfolio

Corresponding author(s): Friedhelm Hummel

Last updated by author(s): 10.04.2024

Reporting Summary

Nature Portfolio wishes to improve the reproducibility of the work that we publish. This form provides structure for consistency and transparency in reporting. For further information on Nature Portfolio policies, see our Editorial Policies and the Editorial Policy Checklist.

Statistics

For all statistical analyses, confirm that the following items are present in the figure legend, table legend, main text, or Methods section.

n/a	Confirmed	
☐	☒	The exact sample size (n) for each experimental group/condition, given as a discrete number and unit of measurement
☐	☒	A statement on whether measurements were taken from distinct samples or whether the same sample was measured repeatedly
☐	☒	The statistical test(s) used AND whether they are one- or two-sided<br>*Only common tests should be described solely by name; describe more complex techniques in the Methods section.*
☐	☒	A description of all covariates tested
☐	☒	A description of any assumptions or corrections, such as tests of normality and adjustment for multiple comparisons
☐	☒	A full description of the statistical parameters including central tendency (e.g. means) or other basic estimates (e.g. regression coefficient) AND variation (e.g. standard deviation) or associated estimates of uncertainty (e.g. confidence intervals)
☐	☒	For null hypothesis testing, the test statistic (e.g. F, t, r) with confidence intervals, effect sizes, degrees of freedom and P value noted<br>*Give P values as exact values whenever suitable.*
☒	☐	For Bayesian analysis, information on the choice of priors and Markov chain Monte Carlo settings
☒	☐	For hierarchical and complex designs, identification of the appropriate level for tests and full reporting of outcomes
☐	☒	Estimates of effect sizes (e.g. Cohen's d, Pearson's r), indicating how they were calculated

Our web collection on statistics for biologists contains articles on many of the points above.

Software and code

Policy information about availability of computer code

Data collection	We used custom-made (Matlab 2018a) scripts to collect the behavioural data
Data analysis	Behaviour: Matlab 2018a<br>MRI data: Statistical Parametric Mapping 12 (SPM12; https://www.fil.ion.ucl.ac.uk/spm/) implemented in MATLAB R2018a (Mathworks, Sherborn, MA, USA), Freesurfer (coded in Bash, 4.4.20(1)-release, and Python (version 3.8.3), https://surfer.nmr.mgh.harvard.edu/); CONN toolbox 2021a (www.nitrc.org/projects/conn, RRID:SCR_009550) running in Matlab 2018a.<br>Both (statistics):<br>R (R Core Team 2021, Vienna, Austria) for linear mixed models: lme4 package (lmer function) , influence.ME package, emmeans package and effectsize packages<br>Matlab R2018a function robustfit for robust linear regressions

For manuscripts utilizing custom algorithms or software that are central to the research but not yet described in published literature, software must be made available to editors and reviewers. We strongly encourage code deposition in a community repository (e.g. GitHub). See the Nature Portfolio guidelines for submitting code & software for further information.

Data

Policy information about availability of data

All manuscripts must include a data availability statement. This statement should provide the following information, where applicable:

- Accession codes, unique identifiers, or web links for publicly available datasets
- A description of any restrictions on data availability
- For clinical datasets or third party data, please ensure that the statement adheres to our policy

Data availability:
All data necessary to generate the main results and figures are available in the Zenodo repository (https://doi.org/10.5281/zenodo.10458885). The Brainnetome atlas was used and can be downloaded from: http://atlas.brainnetome.org/. Tissue properties used for modelling of electric fields were based on the IT'IS Tissue Properties Database v4.0 and can be downloaded here: https://itis.swiss/virtual-population/tissue-properties/overview/

Human research participants

Policy information about studies involving human research participants and Sex and Gender in Research.

Reporting on sex and gender	We recruited a total of 29 women and 19 men. Sex was determined based on self-reporting. We did not consider the factor sex in the analyses.
Population characteristics	We recruited 24 healthy adults (15 women, 25.3 ± 0.1 years old) for the main experiment and 24 other healthy adults (14 women, 24.2 ± 0.5 years old) for the additional experiment
Recruitment	We recruited participants based on platforms for recruitment of subjects and based on previous lists of participants who had performed previous experiments at EPFL. We also distributed flyers at Campus Biotech in Geneva and other public places.<br><br>Selection bias: Healthy young subjects were recruited to a significant part within the university community through verbal or written advertisements. This entails that a disproportionately high number of subjects with a high level of education were recruited. To minimize the impact of this selection bias, the study employed a randomized within-subject design and we distributed the advertisement also at other public places.
Ethics oversight	All participants gave their written informed consent in accordance with the Declaration of Helsinki and the approval of the Cantonal Ethics Committee Vaud, Switzerland (project number 2020-00127).

Note that full information on the approval of the study protocol must also be provided in the manuscript.

Field-specific reporting

Please select the one below that is the best fit for your research. If you are not sure, read the appropriate sections before making your selection.

☒ Life sciences ☐ Behavioural & social sciences ☐ Ecological, evolutionary & environmental sciences

For a reference copy of the document with all sections, see nature.com/documents/nr-reporting-summary-flat.pdf

Life sciences study design

All studies must disclose on these points even when the disclosure is negative.

Sample size	A priori sample size calculation was performed based on effect sizes obtained in our previous behavioral study on reinforcement motor learning (Vassiliadis et al., 2021, iScience) employing a similar paradigm, with a level of significance p < 0.05 (two-sided) and power (1-ß) of 0.90, which resulted in a total sample size of 23 subjects. In order to balance the order of conditions for each subject, we decided to recruit 24 subjects.
Data exclusions	We removed trials from the behavioural analysis in which participants did not react within 1 s after the appearance of the cursor and target, considering that these extremely long preparation times may reflect significant fluctuations in attention.<br>Moreover, to mitigate the impact of isolated influential data points on the outcome of the linear mixed model analyses, we used tools of the influence.ME package (R) to detect and remove influential cases based on Cook's distance: distance > 4 * mean distance. This never removed more than one participant per analysis.
Replication	The main behavioral effects of reinforcement on motor learning (oberved in the tTISSham and tTIS20Hz conditions) were replicated in an additional, independent cohort of 24 participants (See Figure S1b, c, e).
Randomization	All participants performed all experimental conditions. The order of the 6 experimental conditions was pseudo-randomised across participants: the 6 blocks were divided into 3 pairs of blocks with the same tTIS condition and each pair was then composed of one ReinfON and one ReinfOFF block. Within this structure, the order of the tTISTYPE and ReinfTYPE conditions were balanced among the 24 participants.

Blinding	The study was double-blinded. The investigators were not aware of the order of the stimulation type experienced by the participants (tTISSham, tTIS20Hz or tTIS80Hz)

Reporting for specific materials, systems and methods

We require information from authors about some types of materials, experimental systems and methods used in many studies. Here, indicate whether each material, system or method listed is relevant to your study. If you are not sure if a list item applies to your research, read the appropriate section before selecting a response.

Materials & experimental systems

n/a	Involved in the study
☒	☐ Antibodies
☒	☐ Eukaryotic cell lines
☒	☐ Palaeontology and archaeology
☒	☐ Animals and other organisms
☒	☐ Clinical data
☒	☐ Dual use research of concern

Methods

n/a	Involved in the study
☒	☐ ChIP-seq
☒	☐ Flow cytometry
☐	☒ MRI-based neuroimaging

Magnetic resonance imaging

Experimental design

Design type	Task-based fMRI, block design
Design specifications	Each subject performed 6 series of 36 trials in the MRI. One trial lasted 10 seconds. During Training, trials were grouped by bins of 4 and separated by resting periods of 25s.
Behavioral performance measures	We measured the force applied on the MRI-compatible force sensor allowing us to compute the Error relative to the moving target. As explained above, we verified that participants reacted within 1s of the beginning of the trial to make sure that our data was not corrupted by significant fluctuations in attention. Moreover, eye-tracking was also checked during the experiment to verify that participants did not fall asleep in the MRI.

Acquisition

Imaging type(s)	Functional and structural
Field strength	3T
Sequence & imaging parameters	Structural and functional images were acquired using a 3T MAGNETOM PRISMA scanner (Siemens, Erlangen, Germany). T1-weighted images were acquired via the 3D MPRAGE sequence with the following parameters: TR = 2.3 s; TE = 2.96 ms; flip angle = 9°; slices = 192; voxel size = 1 × 1 × 1 mm, FOV = 256 mm; matrix size = 192 x 240 x 256; orientation = sagittal, phase encoding dir = A >> P. Anatomical T2 images were also acquired with the following parameters: TR = 3 s; TE = 409 ms; flip angle = 120°; slices = 208; voxel size = 0.8 × 0.8 × 0.8 mm, FOV = 320 mm; matrix size = 208 x 320 x 320; orientation = sagittal, phase encoding dir = A >> P. Finally, functional images were recorded using Echo-Planar Imaging (EPI) sequences with the following parameters: TR = 1.25 s; TE = 32 ms; flip angle = 58°; slices = 75; voxel size = 2 × 2 × 2 mm; FOV = 112 mm; matrix size = 192 x 240 x 256; orientation = transversal, phase encoding dir = A >> P.
Area of acquisition	Whole-brain

Diffusion MRI ☐ Used ☒ Not used

Preprocessing

Preprocessing software	Structural: the Freesurfer recon-all function was run based on the structural T1w and T2w images (https://surfer.nmr.mgh.harvard.edu/). The BNA parcellation was derived on the individual subject space and the selected ROIs were then co-registered to the functional images and normalised to the MNI space.<br><br>Functional: we analyzed functional imaging data using Statistical Parametric Mapping 12 (SPM12; The Wellcome Department of Cognitive Neurology, London, UK) implemented in MATLAB R2018a (Mathworks, Sherborn, MA). All functional images underwent a common preprocessing including the following steps: slice time correction, spatial realignment to the first image, normalization to the standard MNI space and smoothing with a 6 mm full-width half-maximal Gaussian kernel. T1 anatomical images were then co-registered to the mean functional image and segmented. This allowed to obtain bias-corrected gray and white matter images, by normalizing the functional images via the forward deformation field.
Normalization	SPM normalisation to MNI space, linear and non-linear transformation based on deformation fields obtained from segmentation in SPM.

Normalization template: MNI152 T1, 1mm

Noise and artifact removal: Visual check for co-registration and normalisation. Framewise displacement (FD) was computed and subjects showing more than 40% of time points of FD larger than 0.5mm. No subjects were excluded based on this criterion.

Volume censoring: We did not apply volume censoring

Statistical modeling & inference

Model type and settings: A general linear model was implemented at the single-subject level in order to estimate signal amplitude. Eight regressors were included in the model: 6 head motion parameters (displacement and rotation) and normalised time series within the white matter and the corticospinal fluid.

Effect(s) tested:

Whole brain, 1 sample t-test on the first-level contrasts:
- Sham, ReinfON
- Sham, ReinfON - ReinfOFF

Correlation with behavior - whole brain, multiple regression with individual behavioral results:
- tTIS80Hz vs. tTIS20Hz, ReinfON
- tTIS80Hz vs. tTISSham, ReinfON
- tTIS20Hz vs. tTISSham, ReinfON

Specify type of analysis: ☐ Whole brain ☐ ROI-based ☒ Both

Anatomical location(s):

Number of regions below are based on the Brainnectome atlas (even numbers: right side, odd numbers, left side).

BOLD analysis:
Putamen (225, 226, 229, 230), caudate (219, 220, 227, 228) and NAc (223 and 224)

Effective connectivity:
Motor network: dlPu (229, 230), dCa (227,228), M1 (57 and 58), SMA (9 and 10)
Reward network: NAc (223 and 224), vmPu (225 and 226), vmPFC (41, 45, 47, 49, 187, 42, 46, 48, 50, 188), ACC (77, 179, 183, 178, 180, 184)

Control: Language network as defined by Shirer et al., 2012

Statistic type for inference (See Eklund et al. 2016):

Voxel-wise uncorrected p=0.001 and cluster FDR corrected p=0.05
For activation map during simple condition (Sham, ReinfON): voxel FWE corrected p=0.05 and cluster FDR corrected p=0.05

Correction:

Voxel-wise uncorrected p=0.001 and cluster FDR corrected p=0.05
For activation map during simple condition (Sham, ReinfON): voxel FWE corrected p=0.05 and cluster FDR corrected p=0.05

Models & analysis

n/a	Involved in the study
☐	☒ Functional and/or effective connectivity
☒	☐ Graph analysis
☒	☐ Multivariate modeling or predictive analysis

Functional and/or effective connectivity:

A generalized Psycho-Physiological Interactions (gPPI) connectivity method (from the CONN toolbox) was used to evaluate effective connectivity.
All the following conditions were included in the model:
- Sham, ReinfON
- Sham, ReinfOFF
- tTIS20Hz, ReinfON
- tTIS20Hz, ReinfOFF
- tTIS80Hz, ReinfON
- tTIS80Hz, ReinfOFF
